# Systematic Review on the Efficacy and Safety of Oral Janus Kinase Inhibitors for the Treatment of Atopic Dermatitis

**DOI:** 10.3389/fmed.2021.682547

**Published:** 2021-09-01

**Authors:** Michelle Le, Melissa Berman-Rosa, Feras M. Ghazawi, Marc Bourcier, Loretta Fiorillo, Melinda Gooderham, Lyn Guenther, Sameh Hanna, H. Chih-Ho Hong, Ian Landells, Perla Lansang, Danielle Marcoux, Marni C. Wiseman, Jensen Yeung, Charles Lynde, Ivan V. Litvinov

**Affiliations:** ^1^Division of Dermatology, McGill University Health Centre, Montreal, QC, Canada; ^2^Division of Dermatology, University of Ottawa, Ottawa, ON, Canada; ^3^Faculty of Medicine, University of Sherbrooke, Sherbrooke, QC, Canada; ^4^Division of Pediatric Dermatology, University of Alberta, Edmonton, AB, Canada; ^5^SKiN Centre for Dermatology, Probity Medical Research, and Queens University, Peterborough, ON, Canada; ^6^Division of Dermatology, University of Western Ontario, London, ON, Canada; ^7^Dermatology on Bloor, Toronto, ON, Canada; ^8^Department of Dermatology and Skin Sciences, University of British Columbia, Vancouver, BC, Canada; ^9^Division of Dermatology, Memorial University of Newfoundland, St. John's, NL, Canada; ^10^Division of Dermatology, University of Toronto, Toronto, ON, Canada; ^11^Division of Pediatric Dermatology, University of Montreal, Montreal, QC, Canada; ^12^Section of Dermatology, Department of Medicine, University of Manitoba, Winnipeg, MB, Canada; ^13^Probity Medical Research, Waterloo, ON, Canada

**Keywords:** JAK inhibitor, janus kinase, atopic dermatitis, eczema, abrocitinib, baricitinib, gusacitinib, upadacitinib

## Abstract

**Background:** Atopic dermatitis is a chronic, relapsing and remitting disease that can be difficult to treat despite a recently approved biologic therapy targeting IL-4/IL-13 receptor. Oral janus kinase inhibitors (JAKi) represent a novel therapeutic class of targeted therapy to treat moderate-to-severe atopic dermatitis (AD).

**Objective:** To review the efficacy, safety, and pharmacokinetic characteristics of oral JAKi in the treatment of AD.

**Methods:** A PRISMA systematic review was conducted using MEDLINE, EMBASE (Ovid), and PubMed databases for studies assessing the efficacy, safety, and/or pharmacokinetic properties of oral forms of JAKi in the treatment of AD in pediatric or adult populations from inception to June 2021.

**Results:** 496 papers were reviewed. Of 28 articles that underwent full text screening, 11 met our inclusion criteria for final qualitative review. Four studies examined abrocitinib; three studies examined baricitinib; three examined upadacitinib and one examined gusacitinib (ASN002). Significant clinical efficacy and a reassuring safety profile was reported for all JAKi agents reviewed. Rapid symptom control was reported for abrocitinib, baricitinib and upadacitinib.

**Limitations:** Given the relatively limited evidence for each JAKi and the differences in patient eligibility criteria between studies, the data was not deemed suitable for a meta-analysis at this time.

**Conclusion:** Given their ability to achieve rapid symptom control with a reassuring safety profile, we recommend considering the use of JAKi as a reliable systemic treatment option for adult patients with moderate-to-severe AD, who are unresponsive to topical or skin directed treatments.

## Introduction

Atopic dermatitis (AD), is a chronic and relapsing inflammatory skin condition that affects up to 20% of children and 10% of adults (1). Pruritus is the hallmark of the disease (2); other signs include erythema, scaling, papules, lichenification, excoriations, crusting and vesicles. At times the affected skin can become impetiginized and/or infected with Herpes simplex virus (eczema herpeticum) or molluscum contangiosum (3) leading to increased disease morbidity. Other complications of AD are well-recognized and were reviewed elsewhere (4). In addition to the physical burden, patients with AD have higher rates of psychosocial distress and a reduced quality of life (5–7).

AD is thought to be a multifactorial disease that arises due to both genetic and environmental factors, although the complete pathophysiology has yet to be elucidated (6). Indeed, a meta-analysis of genome-wide association studies, demonstrated that *filaggrin, Inteleukin-13* (*IL-13)*, and *Ovo Like Transcriptional Repressor 1* (*OVOL1)* were found to be the most commonly identified genes associated with an elevated risk of acquiring AD (8). Specifically, IL-13 and OVOL1 regulate filaggrin expression, which is essential for skin barrier protection and plays a crucial role in the pathogenesis of AD. Considering the importance of filaggrin, disruption of the epidermal barrier due to genetic or environmental causes is thought to lead to increased trans-epidermal water loss, making the skin more vulnerable to allergens and pathogen penetration. This, in turn, causes inflammation via the release of chemokines by keratinocytes and subsequent inflammatory cell infiltration (9).

Although historically thought to be a type 2 T helper (Th2) cell driven disease, multiple inflammatory pathways with their respective cytokines have been implicated in AD to varying degrees. These pathways include Th2 (IL-4, IL-5, IL-13, IL-31), Th22 (IL-22), with variable Th1 [interferon (IFN)-γ] and Th17/IL-23 related cytokine involvement (10). These inflammatory pathways have provided potential therapeutic targets for the treatment of AD.

The mainstay for AD management involves the treatment of acute skin flares, management of secondary infections, and prevention of recurrences. General daily skin care for AD includes gentle cleansing of skin, restoration of the skin barrier through the regular use of emollients and avoidance of aggravating factors (11). For acute flares, topical corticosteroids (TCS), topical calcineurin inhibitors (TCI), and/or a topical phosphodiesterase-4 (PDE-4) inhibitor are recommended (12). In moderate-to-severe cases of AD, phototherapy and systemic immunosuppressants (corticosteroids, cyclosporine, azathioprine, mycophenolate mofetil, or methotrexate) can be used (12) although none are approved by Health Canada for the treatment of AD, and side effect profiles for certain systemic immunosuppressants can decrease overall adherence to a treatment plan (11, 13). Access to phototherapy is limited for many patients (14). Although multiple therapeutic modalities are available for the treatment of AD, it remains a challenging disease to manage; in a survey conducted by the National Eczema Association, it was reported that 86% of patients were not satisfied with the treatment of AD (15).

Dupilumab, a monoclonal antibody against IL-4 receptor subunit alpha (Rα) has shown efficacy in many patients and was the first approved biologic therapy for AD that revolutionized the treatment landscape (16). However, approximately 4%-14% experience treatment failure with dupilumab due to either AD worsening (in 5%) or side effects such as conjunctivitis (in 3%) and paradoxical facial erythema (17–19). Patients may also discontinue treatment due to a lack of desired response or for other reasons. Therefore, there remains a need for other targeted therapies. Currently, several other monoclonal antibodies are in phase II/III development and/or pending approval for the treatment of AD, including tralokinumab (20) and lebrikizumab (IL-13 receptor signaling inhibitors), nemolizumab (IL-31 receptor signaling inhibitor) (21–23), and etokimab (IL-33 signaling inhibitor) (24). Given the diversity of cytokines implicated in the inflammatory processes of AD, there is a growing interest toward janus kinases inhibitors (JAKi), which could interfere with the signaling of multiple cytokines simultaneously (25).

**Figure 1:**
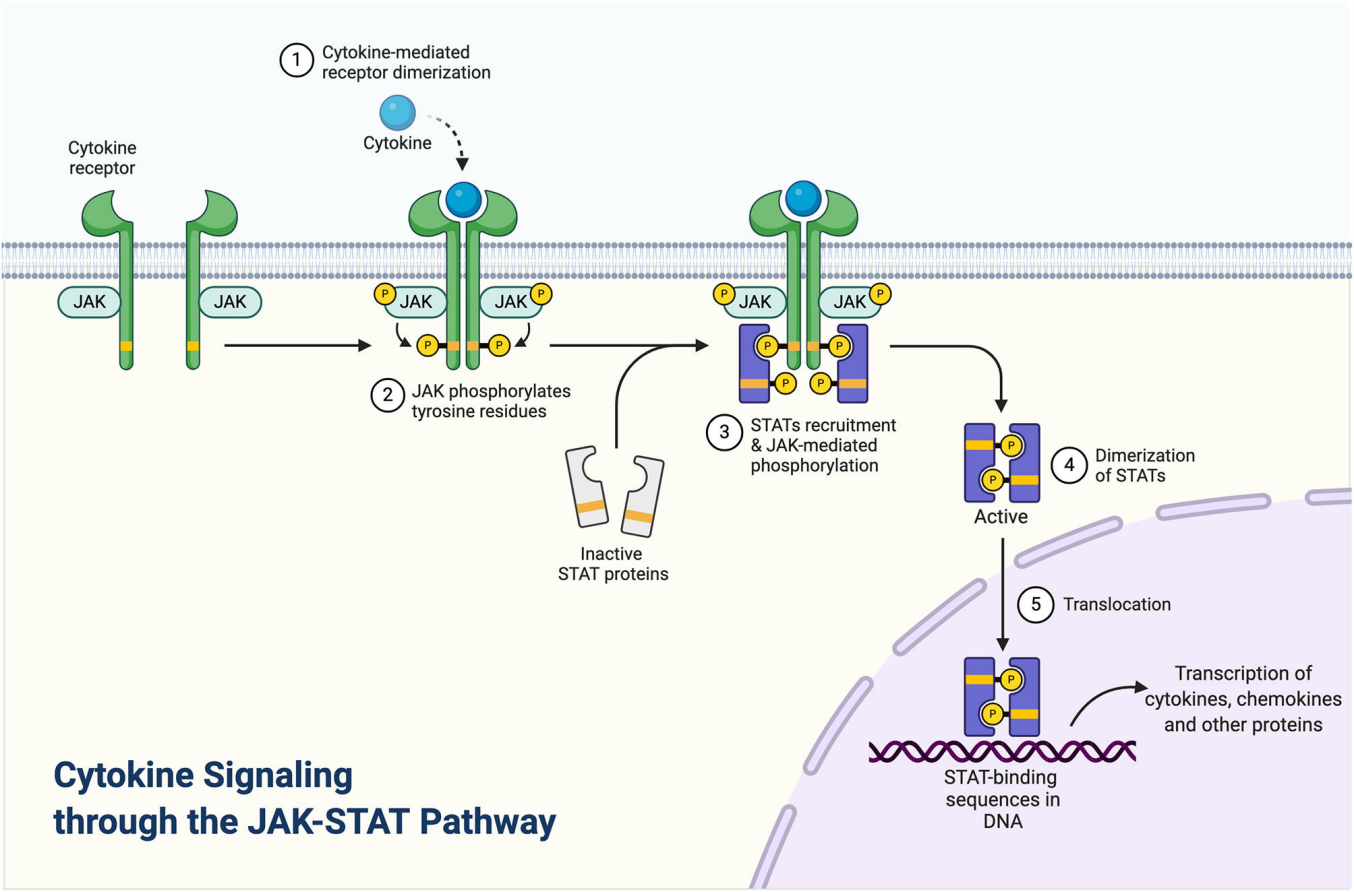
Cytokine signaling through the JAK-STAT pathway. Adapted from “Cytokine signaling through the JAK-STAT pathway,” by BioRender.com (2020). Retrieved from https://app.Biorender.com/biorender-templates.

Janus kinases (JAKs) are signal transduction proteins that are comprised of a family of four proteins: JAK1, JAK2, JAK3, and TYK2 (26). JAKs are recruited to the inflammatory pathways by the binding of cytokines (such as IL-2, IL-4, IL-6, IL-12, IL-21, IL-22, IL-23, or IFN such as IFN- γ) to their cognate receptors that initiate an inflammatory cascade (Figure 1). Recruitment and activation of JAKs results in the phosphorylation of tyrosine residues including residues within the cytokine receptor chains (Figure 1). Consecutively, transcription factors, signal transducer and activator of transcription (STAT) proteins, are recruited and become activated by JAKs phosphorylation. Activated STAT proteins undergo dimerization, which then enables the translocation of these proteins into the nucleus and allows for the transactivation of a broad range of different genes (26, 27). Given that specific JAKs are selectivity activated by different cytokine receptors, this selectivity enables JAKi to demonstrate a defined specificity and different capacities to block cytokine receptor signaling. For instance, while pan-JAKi have a broad inhibitory effect against multiple cytokines, JAKi that selectively target JAK1, JAK2, or TYK2 proteins exclusively, have a more targeted mode of action (28).

In this systematic review, we aim to present the current literature on the pharmacokinetics, clinical efficacy and safety of topical and oral JAKi that were recently approved or are currently under investigation for AD.

## Methods

### Search Strategy

We systematically searched Ovid MEDLINE, EMBASE (Ovid), and PubMed for studies assessing the efficacy, safety, and/or pharmacokinetic properties of oral forms of JAKi in the treatment of AD in pediatric or adult populations from inception to June 2021. We combined free-text search terms for the concept of JAKi (“Janus kinases” OR “JAK inhibitor” or “janus tyrosine kinase inhibitor”) and AD (“atopic dermatitis” or “atopic eczema” or “eczema atopica” or “eczema endogenous” or “eczema infantum” or “eczema, infantile” or “endogenous eczema” or “infantile eczema” or “neurodermatitis constitutionalis” or “neurodermatitis disseminata” or “neurodermatitis, atopic constitutional”). A sample of the search strategy is shown in detail (Supplementary Text 1).

### Study Selection

Two researchers (ML and MB-R) independently assessed study eligibility by title and abstract. When a study was deemed potentially eligible for inclusion, the full text article was obtained and assessed by the reviewers independently. Additional reviewer (FG) was consulted when consensus could not be reached.

We restricted our inclusion criteria to randomized-controlled trials (RCTs) that examined the efficacy or safety of JAKi in the treatment AD as measured by changes from baseline in the Eczema Area and Severity Index (EASI), Investigator's Global Assessment (IGA) score, and the peak pruritus numeric rating scale (PP-NRS). Specifically, proportion of patients achieving EASI-75 and EASI-50, defined as a 75 and 50% reduction from baseline in the EASI score, respectively; achieving an IGA score of 0 or 1 (i.e., clear or almost clear) with an improvement of ≥2 grades from baseline (later referred to as an IGA response); and achieving a PP-NRS score improvement of ≥4-point from baseline (later referred to as PP-NRS response), were required as measures of clinical efficacy. Percentage or absolute value changes in these outcomes were accepted. For safety, qualitative reports of adverse events (AEs) and/or side effects were accepted. We set no restrictions on the concentrations or duration of administrations of experimental compounds. As comparators, we accepted any other type of management for AD, including active surveillance.

We accepted studies published in English or French without date restrictions. Non-randomized trials were excluded. Studies were not included if they were only available as abstracts from conference proceedings or if published in a language other than English or French.

### Quality Assessment and Data Extraction

Two reviewers (ML and MB-R) independently conducted data extraction and methodology quality assessment for all included studies. We extracted the study type, study time frame, type of population (pediatric vs. adult vs. elderly); method of randomization; whether trial was blinded; sample size; follow-up time; the specific JAKi and comparator treatment employed as well as the dosing and regimens; outcome definitions and method for ascertaining treatment effectiveness; and efficacy. For safety, we extracted the most common and most serious treatment emergent adverse events (TEAEs). As a secondary outcome, we extracted the drug's pharmacokinetic characteristics, when reported.

For all studies, the main measures of interest were the efficacy of JAKi on reducing the severity and extent of involvement of AD compared with any other treatments. If reported, efficacy from intention-to-treat analyses (ITT) was preferred and extracted over per-protocol estimates.

We used the modified Cochrane Collaboration tool to assess risk of bias of RCTs (RoB 2) (29), considered the gold-standard for quality assessment of RCTs (30). The tool is structured into five domains through which bias may be introduced into the results: (1) bias arising from the randomization process; (2) bias due to deviations from intended interventions; (3) bias due to missing outcome data; (4) bias in measurement of outcome(s); and (5) bias in the selection of reported results (31). The overall bias assessment within each domain is characterized as “low risk,” “some concerns,” or “high risk of bias,” according to responses provided to the signaling questions within each domain (Supplementary Table 1).

### Data Analysis

We summarized the included reports through descriptive analyses to provide an overview of studies' characteristics, quality, effectiveness, and safety profile of JAKi. Because of the heterogeneity in dosing, length of treatment and length of follow-up, conducting a meta-analysis was not considered. We followed the Preferred Reporting Items for Systematic Reviews and Meta-Analysis (PRISMA) in this systematic review reporting (32).

## Results

### Study Characteristics

**Figure 2:**
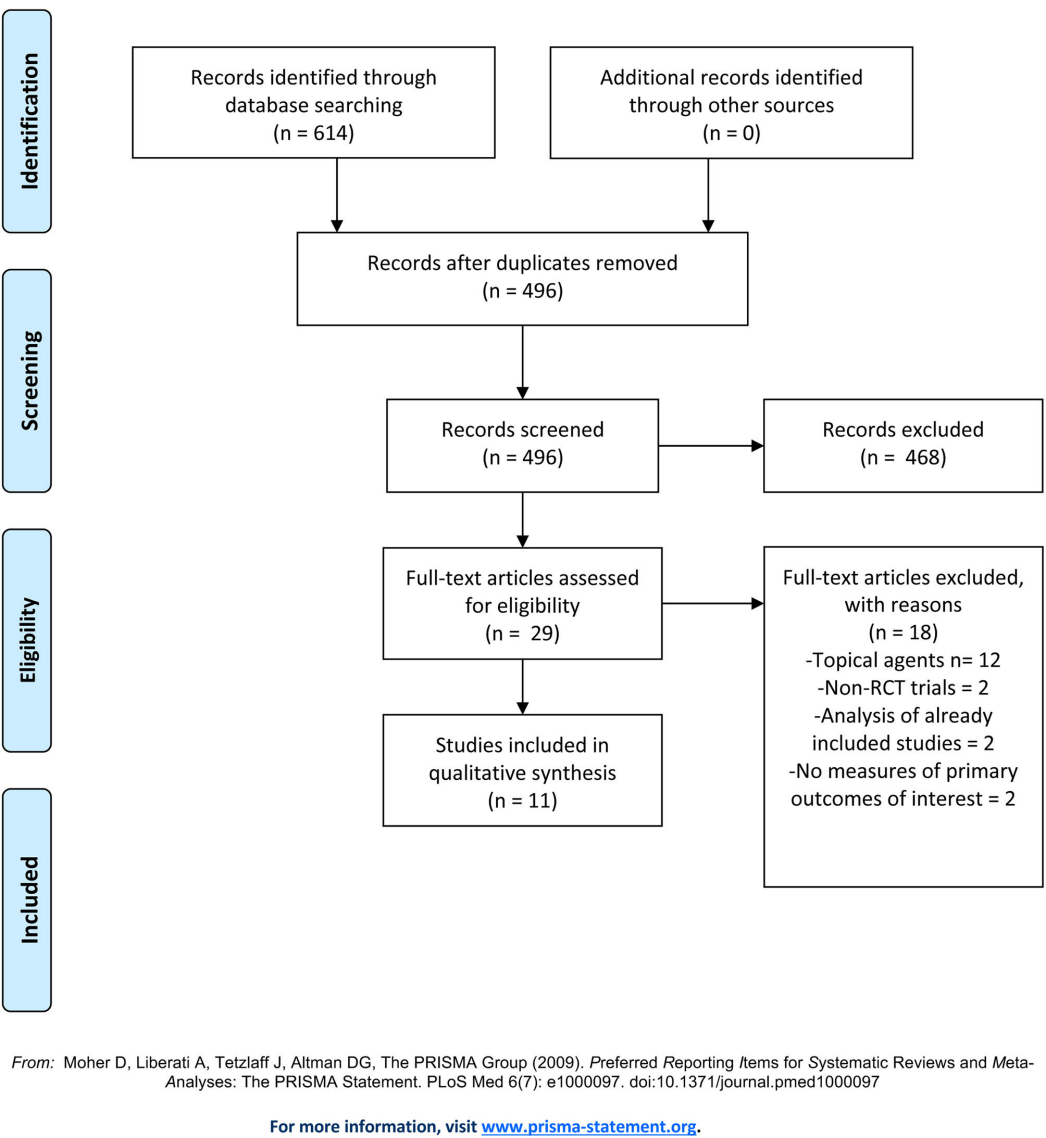
PRISMA Flow Diagram.

Our initial search yielded a total of 614 studies (Figure 2). After removing duplicates, 496 articles remained and were screened by title and abstract. Of the 29 articles that underwent full text screening, 11 met our inclusion criteria. Of these, all examined the efficacy of various JAKi in the treatment of moderate-to-severe AD in adults and adolescents: four examined abrocitinib; three baricitinib; three upadacitinib, and one ASN002. In 8/11 studies inclusion criteria were restricted to patients with moderate-to-severe AD, defined as: an EASI score ≥ 16, IGA score ≥ 3, more than 10% Body Surface Area (BSA) involvement ± a score of ≥ 4 in the PP-NRS (33–38). In the remaining 2 studies, subjects needed to demonstrate an EASI ≥ 12, IGA-score ≥ 3 and BSA involvement > than 10% for inclusion (Table 1). All studies included safety assessment in the form of reported TEAEs (Table 2), and two included evaluation of pharmacokinetics. Physicochemical properties of oral JAK inhibitors are listed in Tables 3, 4.

**Table 1:** Summary of clinical efficacy of published oral JAK inhibitor trials.

**Author**	**Study design**	**Patient eligibility**	**TCS allowed**	**Duration**	**Dose**	***N***	**EASI-75 (%)**	**EASI-50 (%)**	**IGA response (%)**	**PP-NRS response (%)**
Bieber et al. (39)	Multicentre double-blind Phase III RCT	IGA ≥ 3 EASI ≥ 16 BSA ≥ 10% PP-NRS ≥ 4 Failed TCS/TCI or requires systemic therapies for control	Yes	16 weeks	Abrocitinib 100 mg	238	60.3 (*p* < 0.001)	–	34.8 (*p* < 0.001)	47.0
					Abrocitinib 200 mg	226	71.0 (*p* < 0.001)	–	47.5 (*p* < 0.001)	62.8
					Dupilumab 300 mg, every other week	243	65.5	–	38.8	57.1
					Placebo	131	30.6	–	12.9	28.7
Simpson et al. (33)	Multicentre double-blind Phase III RCT	IGA ≥ 3 EASI ≥ 16 BSA ≥ 10% PP-NRS ≥ 4 Failed TCS/TCI or required systemic tx for AD control	No	12 weeks	Abrocitinib 100 mg	158	40 (*p* < 0.0001)	58	24 (*p* = 0.0037)	38 (*p* = 0.0003)
					Abrocitinib 200 mg	155	63 (*p* < 0.0001)	76	44 (*p* < 0.0001)	57 (*p* < 0.0001)
					Placebo	78	12	22	8	15
Silverberg et al. (34)	Multicentre double-blind Phase III RCT	IGA ≥3 EASI≥16 PP-NRS ≥ 4 Failed TCS/TCI or required systemic tx for AD control	No	12 weeks	Abrocitinib 100 mg	158	44.5 (*p* < 0.001)	68.4	28.4 (*p* < 0.001)	55.3
					Abrocitinib 200 mg	155	61.0 (*p* < 0.001)	79.9	38.1 (*p* < 0.001)	45.2
					Placebo	78	10.4	19.5	9.1	11.5
Gooderham et al. (42)	Phase 2b, multicenter, randomized, double-blind, placebo-controlled, parallel-group study	EASI ≥ 12 IGA ≥ 3 BSA ≥ 10% Failed TCS/TCI	No	12 weeks	Abrocitinib 10 mg QD	46	17.4	26.1	10.9	22.7
					Abrocitinib 30 mg QD	45	13.3	33.3	8.9	33.3
					Abrocitinib 100 mg QD	54	40.7 (*p =* 0.004)	55.6^*^	29.6 (*p* < 0.001)	50
					Abrocitinib 200 mg QD	48	64.6 (*p* < 0.001)	79.2^*^	43.8 (*p* < 0.001)	63.6
					Placebo	52	15.4	26.9	5.8	25.5
Simpson et al. (35)	Multicentre double-blind Phase III RCT (Part 1: BREEZE-AD1)	IGA ≥ 3 EASI ≥ 16 BSA ≥ 10% Failed TCS/TCI and/or systemic immunosuppressant therapies	Yes, considered as rescue treatment	16 weeks	Baricitinib 1 mg	127	17.3 (*p* ≤ 0.05)	**–**	11.8 (*p* ≤ 0.05)	10.5
					Baricitinib 2 mg	123	18.7 (*p* ≤ 0.01)	**–**	11.4 (*p* ≤ 0.05)	12.0
					Baricitinib 4 mg	125	24.8 (*p* ≤ 0.001)	**–**	16.8 (*p* ≤ 0.001)	21.5 (*p* ≤ 0.001)
					Placebo	249	8.8	**–**	4.8	7.2
Simpson et al. (35)	Multicentre double-blind Phase III RCT (Part 2: BREEZE-AD2)	IGA ≥ 3 EASI ≥ 16 BSA ≥ 10% Failed TCS/TCI and/or systemic immunosuppressant therapies	Yes, considered as rescue treatment	16 weeks	Baricitinib 1 mg	125	12.8 (*p* ≤ 0.05)	**–**	8.8	6.0
					Baricitinib 2 mg	123	17.9 (*p* ≤ 0.001)	**–**	10.6 (*p* ≤ 0.05)	15.1
					Baricitinib 4 mg	123	21.1 (*p* ≤ 0.001)	**–**	13.8 (*p* ≤ 0.001)	18.7 (*p* ≤ 0.001)
					Placebo	244	6.1	**–**	4.5	4.7
Reich et al. (36)	Double-blind, placebo-controlled, phase 3 RCT	IGA ≥ 3 EASI ≥ 16 BSA ≥ 10% Failed TCS	Yes	16 weeks	Baricitinib 2 mg +TCS	109	43	64 (*p* < 0.001)	24	38
					Baricitinib 4 mg +TCS	111	48 (*p* < 0.001)	70 (*p* < 0.001)	31 (*p* < 0.001)	44 (*p* < 0.001)
					Placebo + TCS	109	23	41	15	20
Guttman-Yassky et al. (43)	Phase 2 parallel, double-blind, placebo-controlled RCT	EASI ≥ 12 BSA ≥ 10% Failed TCS/systemicCS/TCI and immunosuppressants	Yes	16 weeks	Baricitinib 2 mg +TCS	37	30	57	22	–
					Baricitinib 4 mg +TCS	38	34 (*p* = 0.027)	61	21	–
					Placebo + TCS	49	20	37	8	–
Reich et al. (41)	Multicentre, double-blind, placebo-controlled, phase 3 RCT	IGA ≥ 3 EASI ≥ 16 BSA ≥ 10% PP-NRS ≥ 4 Failed TCS/TCI or requires systemic therapies for control	Yes	16 weeks	Upadacitinib 15 mg + TCS	300	64.6 (*p* < 0.0001)	–	40 (*p* < 0.0001)	51.7 (*p* < 0.0001)
					Upadacitinib 30 mg + TCS	297	77.1 (*p* < 0.0001)	–	59 (*p* < 0.0001)	63.9 (*p* < 0.0001)
					Placebo + TCS	304	26.4	–	11	15
Guttman-Yassky et al. (40)	Multicentre, double-blind, placebo-controlled, phase 3 RCT (Part 1: Measure Up 1)	IGA ≥ 3 EASI ≥ 16 BSA ≥ 10% PP-NRS ≥ 4 Failed TCS/TCI or requires systemic therapies for control	Yes, considered as rescue treatment	16 weeks	Upadacitinib 15 mg	281	70 (*p* < 0.0001)	–	48 (*p* < 0.0001)	52 (*p* < 0.0001)
					Upadacitinib 30 mg	285	80 (*p* < 0.0001)	–	62 (*p* < 0.0001)	60 (*p* < 0.0001)
					Placebo	281	16	–	8	12
Guttman-Yassky et al. (40)	Multicentre, double-blind, placebo-controlled, phase 3 RCT (Part 1: Measure Up 1)	IGA ≥ 3 EASI ≥ 16 BSA ≥ 10% PP-NRS ≥ 4 Failed TCS/TCI or requires systemic therapies for control	Yes, considered as rescue treatment	16 weeks	Upadacitinib 15 mg	276	60 (*p* < 0.0001)	–	39 (*p* < 0.0001)	42 (*p* < 0.0001)
					Upadacitinib 30 mg	282	73 (*p* < 0.0001)	–	52 (*p* < 0.0001)	60 (*p* < 0.0001)
					Placebo	278	13 (*p* < 0.0001)	–	5	9
Guttman-Yassky et al. (37)	Phase 2b, double-blind, randomized, parallel-group, dose-ranging trial	IGA ≥ 3 EASI ≥ 16 BSA ≥ 10% Failed TCS/TCI	Not specified	16 weeks	Upadacitinib 7.5 mg QD	42	~28^†^	~48^†^	~11^†^	25^†^
					Upadacitinib 15 mg QD	42	~49^†^	~68	~28^†^	59^†^
					Upadacitinib 30 mg QD	42	~69^†^	~85^†^	~47^†^	55^†^
					Placebo	41	~6^†^	~18^†^	~1^†^	~4^†^
Bissonnette et al. (38)	Phase 1b double-blind, placebo-controlled, RCT	IGA ≥ 3 EASI ≥ 16 BSA ≥ 10% Failed TCS/TCI	Not specified	29 days	Gusacitinib 20 mg	9	0	20	0	–
					Gusacitinib 40 mg	9	71	100 (*p* = 0.003)	43	–
					Gusacitinib 80 mg	9	33	83 (*p* = 0.03)	17	–
					Placebo	9	22	22	11	–

* *Values reached statistical significance, however, p-values were not presented in the publication*.

† *Values were estimated based on figures presented in the publication as exact values were not available*.

*AD, Atopic Dermatitis; BSA, Body Surface Area; CS, Corticosteroids; EASI, Eczema Area and Severity Index; IGA, Investigator's Global Assessment; PP-NRS, peak pruritus numeric rating scale; TCS, Topical Corticosteroids; TCI, Topical Calcineurin Inhibitors; Tx, treatment; RCT, Randomized Controlled Trial*.

**Table 2:** Summary of common TEAEs in published oral JAK inhibitor trials.

**Author**	**Study design**	**Duration**	**Dose**	***N***	**Headache (%)**	**GI symptoms^a^ (%)**	**Respiratory symptoms^b^ (%)**	**Acne (%)**	**AD worsening (%)**	**Cutaneous infections^c^ (%)**	**Elevated Blood Creatinine Phosphokinase (%)**	**Other TEAEs**
Beiber et al. (39)	Multicentre double-blind Phase III RCT	16 weeks	Abrocitinib 100 mg	238	✓ (4.2)	✓ (4.2)	✓ (14.2)	✓ (2.9)	NR	✓ (1.7)	NR	Transient dose-related decreases platelet count was observed in both abrocitinib groups. Most decreases were within normal limits
			Abrocitinib 200 mg	226	✓ (6.6)	✓ (11.1)	✓ (10.6)	✓ (6.6)	NR	✓ (1.8)	✓ (0.4)	
			Dupilumab 300 mg, every other week	243	✓ (5.4)	✓ (2.9)	✓ (13.2)	✓ (1.2)	NR	NR	NR	
			Placebo	131	✓ (4.6)	✓ (1.5)	✓ (11.5)	NR	NR	✓ (0.8)	✓ (0.8)	
Simpson et al. (33)	Multicentre double-blind Phase III RCT	12 weeks	Abrocitinib 100 mg	156	✓ (8)	✓ (9)	✓ (21.8)	NR	✓ (14)	✓ (4.5)	NR	Transient dose-related decreases platelet count was observed in both abrocitinib groups, with a nadir at week 4. Patients in all treatment groups maintained platelet counts within the normal range
			Abrocitinib 200 mg	154	✓ (10)	✓ (20)	✓ (18.8)	NR	✓ (5)	✓ (3.9)	NR	
			Placebo	77	✓ (3)	✓ (3)	✓ (16.9)	NR	✓ (17)	✓ (1.3)	NR	
Silverberg et al. (34)	Multicentre double-blind Phase III RCT	12 weeks	Abrocitinib 100 mg	158	✓ (5.7)	✓ (10.12)	✓ (21.5)	✓ (1.3)	✓ (5.7)	✓ (3.79)	✓ (1.9)	
			Abrocitinib 200 mg	155	✓ (7.7)	✓ (23.2)	✓ (10.9)	✓ (5.8)	✓ (3.9)	✓ (4.5)	✓ (3.2)	
			Placebo	78	✓ (2.6)	✓ (3.84)	✓ (10.2)	0	✓ (15.4)	✓ (5.12)	✓ (2.6)	
Gooderham et al. (42)	Phase IIb, multicenter, randomized, double-blinded, placebo-controlled, parallel-group study	12 weeks	Abrocitinib 10 mg QD	49	✓ (4.1)	✓ (8.2)	✓ (46.9)	NR	✓ (16.3)	NR	NR	Transient decrease in platelet was observed in 30, 100, and 200 mg groups although most decreases were within normal limits
			Abrocitinib 30 mg QD	51	✓ (9.8)	✓ (9.8)	✓ (37.3)	NR	✓ (17.6)	NR	NR	
			Abrocitinib 100 mg QD	56	✓ (8.9)	✓ (10.7)	✓ (42.9)	NR	✓ (14.3)	✓ (1.8)	NR	
			Abrocitinib 200 mg QD	55	✓ (7.3)	✓ (21.8)	✓ (41.8)	NR	✓ (12.7)	NR	NR	
			Placebo	56	✓ (3.6)	✓ (4)	✓ (23.2)	NR	✓ (19.6)	NR	NR	
Simpson et al. (35)	Multicentre double-blind Phase III RCT (Part 1: BREEZE-AD1)	16 weeks	Baricitinib 1 mg	127	✓ (5.5)	✓ (8.7)	✓ (18.1)	NR	NR	✓ (6.3)	✓ (0.8)	
			Baricitinib 2 mg	123	✓ (11.4)	✓ (1.6)	✓ (12.2)	NR	NR	✓ (8.1)	✓ (0.8)	
			Baricitinib 4 mg	125	✓ (8.0)	✓ (8)	✓ (12.8)	NR	NR	✓ (10.4)	✓ (3.2)	
			Placebo	249	✓ (6.4)	✓ (3.6)	✓ (12.9)	NR	NR	✓ (5.6)	✓ (0.8)	
Simpson et al. (35)	Multicentre double-blind Phase III RCT (Part 2: BREEZE-AD2)	16 weeks	Baricitinib 1 mg	125	✓ (4.8)	✓ (4)	✓ (15.2)	NR	NR	✓ (9.6)	✓ (3.2)	
			Baricitinib 2 mg	123	✓ (7.3)	✓ (5.7)	✓ (17.1)	NR	NR	✓ (13)	✓ (0.8)	
			Baricitinib 4 mg	123	✓ (8.9)	✓ (5.7)	✓ (11.4)	NR	NR	✓ (8.9)	✓ (5.7)	
			Placebo	244	✓ (2.0)	✓ (5.7)	✓ (14.3)	NR	NR	✓ (12.3)	✓ (0.4)	
Reich et al. (36)	Multicentre double-blind, placebo-controlled, phase III RCT	16 weeks	Baricitinib 2 mg +TCS	109	NR	✓ (1)	✓ (18)	✓ (1)	NR	✓ (8.3)	✓ (15)	Increased HDL cholesterol levels (≥1.55 mmol/L) observed in 2 mg and 4 mg treatment groups compared to placebo (28, 17, and 10%, respectively)
			Baricitinib 4 mg +TCS	111	NR	✓ (1)	✓ (18.3)	✓ (4)	NR	✓ (11.7)	✓ (22)	
			Placebo + TCS	108	NR	✓ (3)	✓ (13.9)	✓ (1)	NR	✓ (2.8)	✓ (8)	
Guttman-Yassky et al. (43)	Multicentre Phase II parallel, double-blinded, placebo-controlled RCT	16 weeks	Baricitinib 2 mg +TCS	37	✓ (5)	NR	✓ (2.7)	NR	✓ (3)	NR	✓ (3)	
			Baricitinib 4 mg +TCS	38	✓ (13)	NR	✓ (13.2)	NR	NR	✓ (7.9)	✓ (13)	
			Placebo + TCS	49	NR	NR	✓ (4)	NR	✓ (8)	✓ (2)	NR	
Reich et al. (39)	Multicentre, double-blind, placebo-controlled, phase 3 RCT	16 weeks	Upadacitinib 15 mg + TCS	300	✓ (5)	NR	✓ (19)	✓ (10)	✓ (4)	✓ (2)	✓ (4)	Mild-moderate neutropenia observed in 15 and 50 mg treatment groups compared to none in placebo (1, 1% and none, respectively)
			Upadacitinib 30 mg + TCS	297	✓ (5)	NR	✓ (21)	✓ (14)	✓ (1)	✓ (3)	✓ (6)	
			Placebo + TCS	304	✓ (5)	NR	✓ (18)	✓ (2)	✓ (7)	✓ (1)	✓ (2)	
Guttman-Yassky et al. (40)	Multicentre, double-blind, placebo-controlled, phase 3 RCT (Part 1: Measure Up 1)	16 weeks	Upadacitinib 15 mg	281	✓ (5)	NR	✓ (17)	✓ (7)	✓ (3)	✓ (2)	✓ (6)	Transient neutropenia (>500/ microL) observed in 30 mg treatment groups compared to 15 mg treatment and placebo groups (5, 1, and 1%, respectively)
			Upadacitinib 30 mg	285	✓ (7)	NR	✓ (25)	✓ (17)	✓ (1)	✓ (3)	✓ (6)	
			Placebo	281	✓ (4)	NR	✓ (13)	✓ (2)	✓ (9)	✓ (1)	✓ (3)	
Guttman-Yassky et al. (40)	Multicentre, double-blind, placebo-controlled, phase 3 RCT (Part 2: Measure Up 2)	16 weeks	Upadacitinib 15 mg	276	✓ (7)	NR	✓ (13)	✓ (13)	✓ (3)	✓ (3)	✓ (3)	
			Upadacitinib 30 mg	282	✓ (7)	NR	✓ (12)	✓ (15)	✓ (1)	✓ (1)	✓ (4)	
			Placebo	278	✓ (4)	NR	✓ (9)	✓ (2)	✓ (9)	✓ (1)	✓ (2)	
Guttman-Yassky et al. (37)	Multicentre Phase IIb, double-blind, randomized, parallel-group, dose-ranging trial	16 weeks	Upadacitinib 7.5 mg QD	42	✓ (7.1)	✓ (11.9)	✓ (21.4)	✓ (9.5)	✓ (9.5)	NR	NR	Increased frequency of infections were found in treatment groups (41–52%) vs. placebo (20%)
			Upadacitinib 15 mg QD	42	✓ (7.1)	✓ (7.1)	✓ (21.4)	✓ (4.8)	✓ (4.8)	NR	✓ (7.1)	
			Upadacitinib 30 mg QD	42	✓ (9.5)	✓ (7.1)	✓ (19)	✓ (14)	✓ (14)	NR	✓ (9.5)	
			Placebo	40	✓ (2.5)	✓ (7.5)	✓ (12.5)	✓ (2.5)	✓ (5.0)	NR	✓ (5)	
Bissonnette et al. (38)	Multicentre Phase Ib double-blind, placebo-controlled, RCT	29 days	Gusacitinib 20 mg	9	✓ (11)	NR	NR	NR	NR	NR	NR	Mild hypotension observed in one patient receiving 80 mg Gusacitinib
			Gusacitinib 40 mg	9	✓ (44)	✓ (11)	NR	NR	NR	NR	NR	
			Gusacitinib 80 mg	9	✓ (22)	✓ (44)	NR	NR	NR	NR	NR	
			Placebo	9	✓ (33)	✓ (22)	NR	NR	NR	NR	NR	

a *Gastrointestinal symptoms include nausea, vomiting, diarrhea, gastroenteritis, and upper abdominal pain*.

b *Respiratory tract symptoms include upper respiratory tract infections and nasopharyngitis*.

c *Cutaneous infections include viral, fungal bacterial infections including herpes simplex, folliculitis, cellulitis, and tinea. Does not include post-traumatic or post-procedural infections*.

*NR, Not reported; QD, daily; RCT, Randomized Controlled Trial; TEAE, Treatment-Emergent Adverse Event; TCS, Topical Corticosteroids*.

**Table 3:** Summary of physicochemical properties of oral JAK inhibitors.

**JAK inhibitor**	**Molecular formula**	**Molecular weight**	**Lipophilicity (LogP)**
Abrocitinib	C_14_H_21_N_5_O_2_S	323.4	1.24
Baricitinib	C_16_H_17_N_7_O_2_S	371.4	−0.47
Upadacitinib	C_17_H_19_F_3_N_6_O	380.4	2.13
Gusacitinib	C_24_H_28_N_8_O_2_	460.5	1.18

**Table 4:** Summary of half maximal inhibitory concentration (IC50) of oral JAK inhibitors.

**IC50 (nM)**	**JAK1**	**JAK2**	**JAK3**	**TYK2**
Abrocitinib	29	803	–	1,253
Baricitinib	5.9	5.7	560	53
Upadacitinib	43	200	2,300	4,700
Gusacitinib^*^	–	–	–	–

* *Data not reported*.

### Quality Assessment

Overall, we rated six out of eleven studies as low risk of bias (33, 35, 36, 39–41), and the remainder were considered as higher risk (34, 38, 42–44). All included studies employed a central randomization scheme and stipulated blinding of treatment assignment for investigators, patients and study personnel. Post-randomization, two studies (38, 44) had remaining imbalances between groups in IGA-measured-baseline severity of AD and were thus rated as “higher risk of bias” on the “randomization process” domain of the quality assessment tool. Most studies assessed efficacy of treatment by either an ITT analysis, or a modified version of ITT analysis that included all randomized participants who have received at least one-dose of the study's intervention. A single study (38) reported the per-protocol treatment efficacy estimates and was therefore considered at higher risk of bias for the “deviation from intended intervention” element (Supplementary Table 1).

Across all studies, attrition rates were high (9 to 50%, depending on study group) an effect compounded by relatively small sample sizes (range: 36–847). In evaluating the potential effects of missing outcome values on the assessment of treatment efficacy, we considered the stated reasons for attrition within each group. If these reasons differed between groups within a given study, we considered the potential for bias in outcome assessment as “high.” If attrition rates were high but reasons for discontinuation were relatively similar between groups, we deemed the risk of bias in measurement as low. With this reasoning, five studies (34, 38, 42–44) were evaluated as having a high risk for bias in the assessment of treatment efficacy. Since most studies abided by an ITT-analysis for the measurement of efficacy, these missing values likely biased the results toward the null hypothesis of no effect. Therefore, efficacy estimates reported in these studies are considered likely to represent an under-estimation, rather than an over-estimation, of the treatment's true efficacy.

## Abrocitinib

### Clinical Efficacy

Based on *in-vitro* studies, the JAK1 half maximal inhibitory concentration (IC_50_) of abrocitinib is 29 nM (45) (Table 4). The clinical efficacy of abrocitinib, an oral selective JAK1 inhibitor, was studied in four independent clinical trials, of which three were Phase III trials, while one was a Phase II trial (33, 34, 42) (Table 1). In the recent Phase III trial by Bieber et al. (39), a total of 838 adult patients (aged ≥18 years) with moderate-to-severe AD, who failed treatment with TCS or TCI or required systemic therapy to control their disease, were enrolled. Moderate-to-severe AD was defined as IGA ≥ 3, EASI Score ≥ 16, BSA ≥ 10%, and PP-NRS ≥ 4. Patients were randomized in a 2:2:2:1 ratio to 100 mg abrocitinib, 200 mg abrocitinib, 300 mg dupilumab (every other week), or placebo groups and assessed over 16 weeks. While a significantly higher percentage of patients in the 100 and 200 mg abrocitinib groups achieved an EASI-75 score and IGA response at 16 weeks in comparison to a placebo (*p* < 0.001), this significant increase was not noted when comparing outcome measures with the dupilumab treatment group (Table 1). Specifically, an EASI-75 was achieved in 71% of patients in the 200 mg abrocitinib group, 60.3% of the 100 mg abrocitinib group, 65.5% of the dupilumab group and 30.6% of the placebo group at 16 weeks. An IGA response was observed in 47.5% of the 200 mg abrocitinib group, 34.8% of the 100 mg abrocitinib group, 38.8% of the dupilumab group and 12.9% of the placebo group. Interestingly, a significantly higher (*p* < 0.001) proportion of patients achieving a PP-NRS response as early as week 2 was observed in the 200-mg abrocitinib compared to the dupilumab group. However, this response was not observed in the 100 mg abrocitinib group.

In another Phase III JADE MONO-1 trial by Simpson et al. (33) a total of 387 patients (aged ≥12 years) with moderate-to-severe AD, defined as IGA ≥ 3, EASI Score ≥ 16, BSA ≥ 10%, and PP-NRS ≥ 4, were enrolled. Patients were randomized to receive either placebo, 100 or 200 mg of abrocitinib daily for a total treatment duration of 12 weeks. At the end of treatment, the authors found that the proportion of patients who had achieved an IGA response, was significantly higher in the abrocitinib 100 mg group than in the placebo group [37 (24%) of 156 patients vs. six (8%) of 76 patients; *p* = 0.0037], and in the abrocitinib 200 mg group compared with the placebo group [67 [(44%) of 153 patients vs. six (8%) of 76 patients; *p* < 0.0001]. Additionally, the proportion of patients who achieved an EASI-75 response was significantly higher in the abrocitinib 100 mg group [62 (40%) of 156 patients vs. nine (12%) of 76 patients; *p* < 0.0001] and abrocitinib 200 mg group [96 (63%) of 153 patients vs. nine (12%) of 76 patients; *p* < 0.0001]. Interestingly, a significant difference in the proportion of patients achieving a PP-NRS response for 100 mg and 200 mg abrocitinib groups vs. placebo was achieved by the second week of treatment [20, 46, and 3%, respectively, with *p* = 0.0004 (100 mg abrocitinib vs. placebo) and *p* < 0.0001 (200 mg abrocitinib vs. placebo)]. This significant difference in PP-NRS response was maintained at week 12 [38, 57, and 15% of patients in the 100, 200 mg abrocitinib and placebo groups, respectively, with *p* = 0.0003 (100 mg abrocitinib vs. placebo) and *p* < 0.0001 (200 mg abrocitinib vs. placebo)] (Table 1).

Using the same patient inclusion criteria as the JADE MONO-1 trial, Silverberg et al. (34) also examined abrocitinib at 100 and 200 mg concentrations in adults and adolescent patients (12 to 18 years inclusively) with moderate-to-severe AD. A total of 391 patients were randomized to receive either abrocitinib 100 or 200 mg vs. a placebo intervention for 12-weeks duration. Compared to placebo, the proportion of participants achieving an IGA response were 28.7% higher (*p* < 0.001) for the 200 mg group and 19.3% higher (*p* < 0.001) in the 100 mg group. At the end of the 12-weeks, the 200 and 100 mg groups had achieved an EASI-75 response that was 50.5% (*p* < 0.001) and 33.9% (*p* < 0.001) higher than placebo, respectively. Percentage decreases in EASI scores from baseline were greater for both abrocitinib doses than for placebo at all time points. Significant differences in PP-NRS scores between both doses of abrocitinib and placebo were observed by day 2 of treatment, with decreases of 0.7 [95% Confidence Interval (CI), −0.9–0.5] and 0.6 (95% CI, −0.8–0.4) for the 200 mg and 100 mg doses respectively, vs. 0.1 decrease (95% CI, −0.4–0.2) for placebo.

Similar findings of clinical efficacy were demonstrated in the Phase IIb RCT investigating various dosages of abrocitinib vs. placebo in adult patients (≥18 years of age) with moderate-to-severe AD by Gooderham et al. (42) At week 12, 21 of 48 patients receiving 200 mg of abrocitinib (43.8%; *p* < 0.001), 16 of 54 patients receiving 100 mg of abrocitinib (29.6%; *p* < 0.001), and 3 of 52 patients receiving placebo (5.8%) achieved an IGA response. Additionally, through logistic regression modeling, authors estimated that a greater proportion of patients achieved an EASI-75 response in the 200 mg [estimated 31 of 48 (63.7%), *p* < 0.001] and 100 mg [estimated 22 of 54 (41.6%), *p* = 0.004] groups when compared to a placebo group [estimated 8 of 52 (15.6%)]. Significant differences from placebo in percentage reduction in EASI score from baseline were observed as early as week 1 (first postbaseline assessment) in the 200 mg group [least squares mean (LSM) difference from placebo, −28.3%; *p* < 0.001], and at week 2 in the 100 mg group (−14.9%; *p* = 0.03). Decreases from baseline in EASI score for the 200 mg and 100 mg groups were found to plateau by weeks 4 to 6 and were maintained through week 12.

### Safety

In the four trials (33, 34, 39, 42), gastrointestinal and respiratory symptoms were found to be the most frequently reported TEAEs in the abrocitinib 100 and 200 mg groups, followed by a headache. AD worsening was found to be more common in placebo compared to abrocitinib groups. Moreover, in all four trials, transient dose-related numeric decreases in median platelet count were observed in patients receiving abrocitinib, with a nadir observed at week 4 and a return toward baseline values thereafter. Nevertheless, the majority of patients in treatment groups maintained platelet counts within the normal range (Table 2).

Specifically, in the Bieber et al. (39) trial, nausea was the most frequently reported TEAE in each of the 100 mg, 200 mg abrocinitib and dupilumab groups. Mild to moderate acne was also more frequently reported in abrocitinib groups (6.6 and 2.9% for 200 and 100 mg abrocitinib, respectively), in comparison to dupilumab or placebo groups (1.2 and 0%, respectively) (Table 2). Two malignancies were reported in this study: one cutaneous squamous-cell carcinoma in the in the 200-mg abrocitinib group, and one invasive intraductal breast neoplasia in the dupilumab group. The authors did not comment whether or not these malignancies were considered to be treatment-related. No deaths, or venous thromboembolisms (VTEs) were observed during this trial.

In the Phase III JADE MONO-1 trial (33), the most frequently reported TEAE in the abrocitinib 100 mg and 200 mg groups were nausea (9% in 100 mg and 20% in 200 mg groups) and nasopharyngitis (15% in 100 mg and 12% in 200 mg groups). Other common TEAEs included headache, and upper respiratory tract infection (URTI) symptoms (≥5% in any treatment group). Herpes virus infections were reported in all treatment groups, albeit uncommon [one (<1%) of 156 patients in the abrocitinib 100 mg group, and three (~2%) of 154 patients in the abrocitinib 200 mg group].

Serious adverse events (SAEs) were reported in five (3%) of 156 patients in the abrocitinib 100 mg group, five (3%) of 154 patients in the abrocitinib 200 mg group, and three (4%) of 77 patients in the placebo group. Among these patients, only two SAEs were considered treatment-related: in one patient receiving the abrocitinib 200 mg, who developed chronic inflammatory bowel disease, abrocitinib was permanently discontinued leading to full recovery; the other patient was in the abrocitinib 100 mg group and developed acute pancreatitis during the treatment period. Thus, abrocitinib was permanently discontinued, and the patient recovered. In this study, no cases of VTE, malignancies, major adverse cardiovascular events, changes in blood creatinine phosphokinase (CPK) levels or deaths were observed.

In Silverberg's Phase III trial of abrocitinib in adults and adolescents (34), the most frequent treatment TEAEs of any causality included nausea in the 200 mg group (14.2%), nasopharyngitis in the 100 mg group (12.7%) and worsening AD in the placebo group (15.4%). Other TEAEs of interest were acne (5.8% in the 200 mg group, 1.3% in 100 mg group and none in placebo group), folliculitis (3.2% in in 200 mg group and 2.6% in placebo group), vomiting (5.2% in 200 mg group, 1.3% in both 100 mg and placebo groups), and upper abdominal pain (3.9% in 200 mg vs. 1.3% and 0 in 100 mg and placebo respectively). SAEs that were considered related to treatment were reported for two patients in the 100 mg group (herpangina and pneumonia) and two patients in the placebo group (eczema herpeticum and a case of staphylococcal infection). None were observed in the 200 mg group. An elderly participant with pre-existing aortic valve sclerosis and untreated hypertension experienced a sudden cardiac death 3-weeks after discontinuation of abrocitinib. The event was not considered related to treatment. Furthermore, no cases of thromboembolisms or malignant neoplasms were reported in any treatment groups. The authors also reported a dose-related increase of ~10% in high-and-low-density lipoprotein levels, as well as an increase in CPK levels, for both the 200 mg and 100 mg groups compared to placebo (34).

The most frequently reported of TEAEs (≥3 patients in any treatment group) in Gooderham et al. Phase IIb study of abrocitinib included diarrhea, nausea, viral URTI, headache, and worsening atopic dermatitis (42). Two of 267 patients experienced SAEs that were considered related to treatment; one patient in the 200 mg group developed pneumonia during follow-up after initiation of cyclosporine, which was continued, and treated with antibiotics; and one patient in the 100 mg group developed eczema herpeticum during the treatment period, abrocitinib was permanently discontinued. One patient in the 200 mg group reported a pulmonary embolism (PE) after traveling a long distance by car with baseline laboratory values within normal limits. One patient receiving 10mg dose developed a melanoma, which was deemed not related to treatment. No treatment-related trends in serum lipids and transaminase levels were observed in the trial. CPK levels were, unfortunately, not reported.

## Baricitinib

### Clinical Efficacy

Baricitinib is an oral selective JAK1 and JAK2 inhibitor that blocks the downstream action of several cytokines in AD pathogenesis, including thymic stromal lymphopoietin, IL-4, IL-5, IL-13, IL-22, and IL-31 (36). According to *in-vitro* analyses, the baricitinib IC_50_ values were reported as 5.9 and 5.7 nM for JAK1 and JAK2 inhibition, respectively (46) (Table 4). In the 16-week Phase III independent BREEZE-AD1, BREEZE-AD2 trials by Simpson et al. (35) the efficacy of baricitinib vs. placebo was assessed in a total of 1,239 adult patients with moderate-to-severe AD at varying doses. In both BREEZE-AD1 and BREEZE-AD2 studies, moderate-to-severe AD was defined as IGA ≥ 3, EASI ≥ 16, BSA ≥ 10%. Eligible patients also had to demonstrate an inadequate response to TCS/TCIs and/or systemic immunosuppressant therapies. In total, 624 patients were enrolled in BREEZE-AD1, where they were randomized to daily placebo (*n* = 249), 1 mg (*n* = 127), 2 mg (*n* = 123), or 4 mg (*n* = 125) baricitinib groups. Similarly, a total of 615 patients were enrolled in BREEZE-AD2, where patients were randomized to similar groups [placebo (*n* = 244), 1 mg (*n* = 125), 2 mg (*n* = 123), or 4 mg (*n* = 123)].

In both trials, 2 mg and 4 mg of baricitinib achieved the study's primary efficacy outcome: a significant improvement vs. placebo for the proportion of patients achieving an IGA response at week 16. The percentage of patients achieving IGA response was 4.8% for placebo, 11.4% for baricitinib 2 mg, and 16.8% for baricitinib 4 mg (baricitinib 2 mg, *p* ≤ 0.05; baricitinib 4 mg, *p* ≤ 0.001 vs. placebo) in BREEZE-AD1, and 4.5% for placebo, 10.6% for baricitinib 2 mg, and 13.8% for baricitinib 4 mg (baricitinib 2 mg, *p* ≤ 0.05; baricitinib 4 mg, *p* ≤ 0.001 vs. placebo) in BREEZE-AD2 (Table 1).

In both studies, 4 mg baricitinib treatment was found to lead to significant improvement for all secondary study endpoints, including a significantly higher proportion of patients achieving a PP-NRS response compared to placebo (*p* ≤ 0.001) at weeks 1, 2, 4, and 16; proportion of patients achieving EASI-75; and percentage change from baseline EASI score. Similarly, 2 mg baricitinib treatment also demonstrated a significant improvement for the aforementioned secondary endpoints in both trials, except for the proportion of patients achieving a PP-NRS response compared to placebo at week 1. However, 1 mg of baricitinib treatment led to inconsistent clinical outcomes in primary and secondary endpoints in both trials.

In another Phase III clinical trial by Reich et al. (36) (BREEZE-AD7), a total of 329 patients with moderate-to-severe AD were randomly assigned (1:1:1) to receive 2 mg of baricitinib once daily (*n* = 109), 4 mg of baricitinib once daily (*n* = 111), or placebo (*n* = 109) for 16 weeks. The use of low-to-moderate potency TCSs as well as TCIs and crisaborole for active lesions was allowed throughout the trial. Rescue therapy with high- or ultrahigh-potency TCSs or systemic therapies were available for patients who experienced worsening and unacceptable AD symptoms after 2 weeks of treatment.

The proportion of patients who achieved the primary endpoint of IGA response at week 16 was significantly higher for patients treated with 4 mg of baricitinib *vs*. placebo [34 of 111 (31%); *p* = 0.004]. Unlike the BREEZE-AD1 and BREEZE-AD2 trials, the primary end point for 2 mg of baricitinib was not met [26 of 109 (24%); *P* = 0.08]. As such, secondary endpoints were only evaluated for the 4 mg of baricitinib group. Specifically, the 4 mg of baricitinib group experienced a significant improvement compared with the placebo group (*p* < 0.001) for a proportion of patients, who achieved an EASI-75 response at week 16 [53 of 111 (48%) in the 4 mg group, vs. 25 of 109 (23%) in the placebo group], proportion of patients who achieved a PP-NRS response at week 4 [52 of 100 (52%) for the 4 mg group vs. 11 of 104 (11%) for the placebo group] and week 16 [44 of 100 (44%) for the 4 mg group, 37 of 97 (38%) vs. 21 of 104 (20%) for the placebo group] (Table 1).

Findings of a Phase II RCT investigating the clinical efficacy of 2 mg and 4 mg baricitinib vs. placebo in adult patients with moderate-to-severe AD by Guttman-Yassky et al. (43) demonstrated similar results. In this study, however, moderate-to-severe AD was defined by EASI ≥ 12, BSA ≥ 10% and eligible patients had to fail treatment with either TCS, TCI, systemic corticosteroids or other conventional immunosuppressants. Additionally, triamcinolone 0.1% cream was used throughout the study according to label instructions or as recommended by the investigator. Significantly more patients who received 4 mg baricitinib, achieved EASI-50 than did patients who were assigned to a placebo arm [61 vs. 37% (*p* = 0.027)] at 16 weeks. However, the proportion of patients achieving EASI-50 in the 2 mg baricitinib group did not reach statistical significance at 16 weeks (*p* = 0.065).

### Safety

Overall, the most common TEAEs reported in all baricitinib studies reviewed were respiratory symptoms, headache, cutaneous infections, gastrointestinal symptoms and elevation of blood CPK. Specifically, in the study conducted by Simpson et al. (35), the most frequently reported TEAEs (>2% in any treatment group) were nasopharyngitis, URTIs, CPK elevations and headaches. However, there was no increase in the frequency of nasopharyngitis and URTIs, when comparing baricitinib with placebo. Headaches were reported at similar rates in patients treated with 4 mg baricitinib and placebo in BREEZE-AD1 (8.0 and 8.9%, respectively), while a greater percentage of patients reported headaches in the 4 mg baricitinib group compared to placebo in the BREEZE-AD2 trial (6.4 and 2.0%, respectively). Nevertheless, reported headaches were mild (76% of reported cases) and short-lived (median duration of ≤ 5 days), with none requiring study-drug interruption or discontinuation. Herpes simplex was also observed more frequently with baricitinib in BREEZE-AD1 trail. Most cases were of mild or moderate severity in both studies and did not cause SAEs or required drug discontinuation. Although CPK elevations were common, most cases (16 of 20) were asymptomatic and either resolved to below the upper limit of normal or were resolving during the study without treatment interruptions. Three patients treated with baricitinib had temporary treatment interruption with resolution of CPK elevations and one patient discontinued the study. No changes in serum lipids were reported. No deaths or VTEs (including PE and Deep Venous Thrombosis [DVT]) were reported in any group. There were no malignancies reported in baricitinib treatment groups.

In the BREEZE-AD7 trial, the most frequently reported (≥2% in any treatment group) TEAEs for 4 mg and 2 mg baricitinib doses compared with placebo were nasopharyngitis, folliculitis, oral herpes, URTI, acne, diarrhea, and back pain (36). One 51-year-old female patient in the 4 mg baricitinib group experienced a PE in the context of receiving oral contraceptives and having a previous history of smoking (7 pack-years). The patient subsequently discontinued treatment and recovered from the event. No major adverse cardiovascular events, malignant tumors, or deaths were reported.

CPK levels were elevated with baricitinib compared with placebo, with most increases being classified as Common Terminology Criteria for Adverse Events (CTCAE) grades 1 and 2 (increase of CPK of <2.5 times and 2.5–5 times the upper limit of normal, respectively) (36). Additionally, CPK elevations were not associated with evidence of muscle injury (e.g., rhabdomyolysis). Although changes were seen in lipid levels, including increases in high-density lipoprotein (HDL) level (≥60 mg/dL; 4 mg group, 28%; 2 mg group, 17%; and placebo group, 10%), changes in low-density lipoprotein (LDL) levels were similar in all groups (≥160 mg/dL; 4 mg group, 3%; 2 mg group, 3%; and placebo group, 4%).

Similarly, in the phase II trial by Guttman-Yassky et al. headaches and nasopharyngitis were reported as common TEAEs (43). Additionally, it was noted that infections were not increased in the groups treated with 2 mg or 4 mg baricitinib compared with those who received a placebo. In both 2 mg and 4 mg baricitinib-plus-TCS groups, the authors observed asymptomatic increases in CPK levels of ≥ 30 U/L at week 16 (43). No deaths, VTEs or malignancies were reported.

## Upadacitinib

### Clinical Efficacy

The clinical efficacy of upadacitinib, a selective JAK1 inhibitor, has recently been determined by two Phase III studies by Reich et al. (41) and Guttman-Yassky et al. (40). In the study by Reich et al. (41) the efficacy of upadacitinib at 15 and 30 mg daily with TCS vs. placebo with TCS was assessed in 901 adolescent (aged 12 to 17 years old) and adult (aged 18 to 75 years) patients with moderate to severe atopic dermatitis, as defined by the Hanefin and Rajka criteria. At 16 weeks, authors found that the proportion of patients who had achieved an EASI-75, was significantly higher in the 15 mg and 30 mg upadacitinib with TCS treatment groups than in the placebo with TCS group (64.6, 77.1, and 26.4%, respectively; *p* < 0.0001; Table 1). Additionally, a significantly higher proportion of patients achieved an IGA response at week 16 in both 15 and 30 mg upadacinitib with TCS treatment groups in comparison to placebo alone (Table 1).

The proportion of patients achieving a PP-NRS response as early as 1 week was significantly higher in patients receiving 15 and 30 mg upadacitinib with TCS treatments than in the placebo with TCS treated group (12.2, 19.2, 3.1%, respectively; *p* < 0.0001). A similar trend was noted in the proportion of patients achieving an EASI-75 score at 2 weeks. Reich et al. (41) documented 31.0% of patients achieving an EASI-75 score in the 15 mg upadacitinib with TCS group, 44.1% in the 30 mg upadacitinib with TCS group, and 6.9 % in the placebo with TCS group treatment (*p* < 0.0001 when comparing 15 and 30 mg upadacitinib with TCS treatment groups *vs*. placebo).

In the study by Guttman-Yassky et al. (40), the efficacy of upadacitinib at 15 and 30 mg daily vs. placebo were assessed in Measure Up 1 and Measure Up 2 replicate Phase III RCTs. A total of 1,683 adolescent (aged 12 to 17 years old) and adult (aged 18 to 75 years) patients with moderate-to-severe AD, defined as IGA ≥ 3, EASI ≥ 16, BSA ≥ 10%, and PP-NRS ≥ 4 were enrolled in both studies.

Eight hundred forty-seven patients participated in Measure Up 1, where they were Randomized to daily placebo (*n* = 281), 15 mg (*n* = 281, or 30 mg (*n* = 285) upadacitinib treatment groups. Eight hundred thirty-six patients participated in Measure Up 2, where patients were randomized to placebo (*n* = 278), 15 mg (*n* = 276), or 30 mg (*n* = 282) upadacitinib treatment groups.

In both Measure Up 1 and 2 trials, patients in the 15 and 30 mg upadacitinib groups demonstrated important efficacy outcomes. Namely, the study showed a significantly higher (*p* < 0.0001 in all cases) proportion of patients in the 15 or 30 mg of upadacitinib groups achieving an EASI-75 score (coprimary endpoint), IGA response (coprimary endpoint) and PP-NRS at week 16 vs. placebo (Table 1).

Interestingly, a significantly higher proportion (*p* < 0.0001) of patients in both 15 mg and 30 mg upadacitinib treatment groups achieved a PP-NRS response as early as 1 week in comparison to placebo, in the Measure Up 1 and Measure Up 2 Trials (Measure Up 1: 15.0% in the 15 mg upadacitinib group, 19.6% in the 30 mg group, and 0.4% in the placebo group; Measure Up 2: 7.4% in the 15 mg upadacitinib group, 15.7% in the 30 mg group, and 3.6% in the placebo group). Similarly, a significantly higher proportion (*p* < 0.0001) of patients in both 15 mg and 30 mg upadacitinib treatment groups achieved an EASI-75 score as early as 2 weeks in comparison to placebo, in the Measure Up 1 and Measure Up 2 Trials (Measure Up 1: 38.1% in the 15 mg upadacitinib group, 47.4% in the 30 mg group, and 3.6% in the placebo group; Measure Up 2: 33.0% in the 15 mg upadacitinib group, 44.0% in the 30 mg group, and 3.6% in the placebo group).

In another recent study by Guttman-Yassky et al. (37), the clinical efficacy of the selective JAK 1 inhibitor, upadacitinib, was investigated over a period of 16 weeks in this Phase IIb, double-blinded, randomized, parallel-group, dose-ranging trial. Patients with moderate-to-severe AD, defined by IGA ≥ 3, EASI ≥ 16, BSA ≥ 10%, and who failed treatment with TCSs/TCIs were randomized 1:1:1:1 to once-daily upadacitinib oral monotherapy 7.5, 15, or 30 mg or placebo groups. Results at 16 weeks demonstrated that EASI-50, EASI-75, and EASI-90 responses were also achieved at week 16. EASI-100 was achieved by 2.4% (1 of 42; *p* = 0.43), 9.5% (4 of 42; *P* = 0.05), and 24% (10 of 42; *p* = 0.001) of patients in the upadacitinib 7.5-, 15-, and 30 mg groups, respectively, vs. none (0 of 41) in the placebo group. Each upadacitinib dose level was significantly superior to placebo for achieving an IGA response and patient assessment of pruritus (achievement of PP-NRS response) at week 16. Interestingly, efficacy at the studied doses was generally demonstrated by weeks 1 to 4, with peak values reached and maintained after weeks 4 or 8.

### Pharmacokinetics

In the study by Guttman-Yassky et al. (37), pharmacokinetic measures were investigated, where it was found that upadacitinib exposures were approximately dose proportional over the 7.5- to 30 mg dose range. Upadacitinib median (interquartile range) plasma concentrations around peak and trough periods were consistent with exposures previously observed for the evaluated doses in healthy volunteers [7.5 mg dose: 10.6 (0.8–21.0) and 2.8 (1.4–4.5) ng/mL, respectively; 15 mg dose: 32.5 (22.7–39.3) and 3.6 (1.8–7.0) ng/mL; 30 mg dose: 57.0 (28.1–94.8) and 8.1 (6.6–16.6) ng/mL] (37).

### Safety

In both the Reich et al. (41) and Guttman-Yassky et al. (40) Phase III studies, TEAEs were reported more frequently in the upadacitinib treatment groups than the placebo group (Table 2). The most common reported TEAEs (>5% in any treatment group) were acne, respiratory symptoms, headache, elevation in CPK and worsening of AD. The majority of patients who reported treatment-emergent acne had mild to moderate symptoms consisting of inflammatory papules, pustules, comedones, with few cysts and nodules. While in the Reich et al. (41) study, none of the acne events were considered as severe and did not lead to treatment discontinuation, in the Guttman-Yassky et al. (40) study, one acne event was severe, involving >30% of body surface area. Additionally, among patients in the Measure up 1 and 2 trials, one patient in the upadacitinib 15 mg group and one patient in the upadacitinib 30 mg group discontinued study drug because of moderate acne.

With respect to potentially clinically important laboratory findings, most reports of elevations in CPK levels were asymptomatic and associated with exercise. In the Reich et al. (41) study, the elevated CPK levels were reported to be dose related. In the Guttman-Yassky et al. (40) study, only one case of elevated creatinine phosphokinase levels was reported in the upadacitinib 15 mg group, which led to treatment discontinuation. Transient, mild to moderate, neutropenia was also observed more frequently in upadacitinib groups in comparison to placebo in both Phase III studies. Only one event, occurring in the Reich et al., study lead to discontinuation of 30 mg upadacitinib treatment.

No treatment-related deaths or VTEs were reported in the upadacinitib groups in both Phase III RCTs. In the Reich et al. (41) study, two malignancies were reported in the upadacitinib 30 mg with TCS treatment group: one non-melanoma skin cancer (a keratoacanthoma) identified on treatment day 45 and one adenocarcinoma of the colon identified on treatment day 7. The case of colon adenocarcinoma was considered as a non-treatment-related, serious adverse event that lead to the discontinuation of upacitinib. In the Guttman Yassky et al. (40) study, six cases of malignancy were reported in the upadacitinib groups, all of which were determined not to be treatment-related [squamous cell skin carcinoma (*n* = 2), basal cell skin carcinoma (*n* = 1), breast cancer (*n* = 1), gastric cancer (*n* = 1), and anal cancer (*n* = 1)].

In the Guttman-Yassky et al. (37) Phase IIb study, TEAEs were reported in 71% (30 of 42), 74% (31 of 42), and 79% (33 of 42) of patients receiving upadacitinib 7.5, 15, and 30 mg, respectively, vs. 63% (25 of 40) of patients receiving a placebo (Table 2). The most frequently reported TEAEs were gastrointestinal and URTI symptoms, followed by acne and AD worsening, all of which were reported as mild or moderate in severity. Additionally, in the 15 mg and 30 mg groups, increased blood CPK was observed in 7.1 and 9.5%, respectively. Nevertheless, the CPK elevations were asymptomatic in patients receiving upadacitinib and reported to be mild to moderate in severity. There was no relationship noted between the dose of upadacitinib and the occurrence of particular TEAEs.

Two patients in the upadacitinib 7.5 mg group had SAEs, namely worsening AD (skin infection and exacerbation of AD) in the context of contact dermatitis and a lower jaw pericoronitis due to recurring tooth infections, not thought to be associated with the treatment. One patient in the upadacitinib 15 mg group had appendicitis. All SAEs in patients who received upadacitinib resolved with treatment.

There were no deaths, opportunistic infections, malignancies, gastrointestinal perforations, herpes zoster, renal dysfunction, active or latent tuberculosis reactivation, adjudicated cardiovascular events, or VTEs. While infections were more common with upadacitinib than with placebo, there were fewer serious infections with upadacitinib.

## Gusacitinib

### Clinical Efficacy

Bissonnette et al. (38) were the first to demonstrate gusacitinib (ASN002) as an effective AD treatment in a double-blinded, placebo-controlled Phase Ib RCT. Gusacitinib is an oral dual inhibitor of JAK and tyrosine-protein kinase SYK (also known as spleen tyrosine kinase). Patients included had a diagnosis of moderate-to-severe AD defined by an IGA ≥ 3, EASI ≥ 16, BSA ≥ 10%. In this study, 36 patients were randomized at a 3:1 ratio, gusacitinib or placebo, whereby 9 patients were included in the 20, 40, and 80 mg gusacitinib once daily and placebo groups. Each patient received either gusacitinib or placebo once daily for 28 days. In the context of our systematic review, clinical efficacy was determined via EASI-50 and EASI-75 tools over 29 days. Gusacitinib was found to be significantly superior to placebo for the proportion of patients achieving EASI-50 in the 40 mg and 80 mg dose groups at end of treatment (*p* = 0.003 and *p* = 0.03, respectively) (Table 1). However, the same efficacy could not be demonstrated in the 20 mg gusacitinib group. The proportion of patients achieving EASI-75 was also greater in the 40 and 80 mg gusacitinib groups compared to placebo, although the difference did not reach statistical significance (*p* = 0.06 and *p* = 0.65, respectively).

### Pharmacokinetics

The trial by Bissonnette et al. (38) also reported the pharmacokinetic parameters of gusacitinib in AD patients (38). For 20 mg gusacitinib, the mean maximum concentration (C_max_) was found to be 67.8 ng/ml, and half-life of 6.62 h. For 40 mg gusacitinib the mean C_max_ was found to be 136 ng/ml, and half-life of 9.10 h. For 80 mg gusacitinib the mean C_max_ was found to be 186 ng/ml, and half-life of 11.2 h.

### Safety

The most common TEAEs were headache and nausea in 7 and 5% of patients who received gusacitinib, respectively (Table 2) (38). There were 2 TEAEs that led to discontinuation including a subject with mild hypertension and another with low lymphocyte counts. The event of mild hypertension was reported in a patient receiving 80 mg gusacitinib and was classified as being possibly related to treatment. The patient with lymphopenia had had low pre-treatment lymphocyte levels and the AE was not considered to be related to treatment. No clinically significant changes in lipid profile were observed in the study. CPK levels were not reported in this trial. Additionally, no VTEs, malignancies or deaths were noted (38).

## Discussion

AD is a common and debilitating inflammatory skin disease driven by barrier dysfunction and abnormal Th cell activation. Multiple inflammatory pathways and their respective cytokines are believed to be involved in the chronicity and relapsing nature of the disease. The JAK-STAT and SYK pathways have been shown to assert a downstream modulating effect on AD-associated cytokines. Therefore, JAKi have introduced a promising novel area of therapeutics in the treatment of AD, as well as in other cytokine-mediated autoimmune and inflammatory diseases. With the growing number of clinical trials evaluating the efficacy of various JAKi for the treatment of AD, we sought to systematically review the literature to synthesize and evaluate the available evidence on efficacy and safety of these new compounds. We identified 11 RCTs evaluating the efficacy and safety of four compounds: abrocitinib, baricitinib, upadacitinib, and gusacitinib. A summary of the physicochemical properties of JAKi discussed are provided in Table 3. Given the relative paucity of evidence for each individual compound and the differences in patient eligibility criteria among studies, the data was not deemed suitable for a meta-analysis at this time. Nevertheless, the presented review provides a comprehensive summary of the evidence, most of which lends support to the use of JAKi in the treatment of AD.

**Figure 3:**
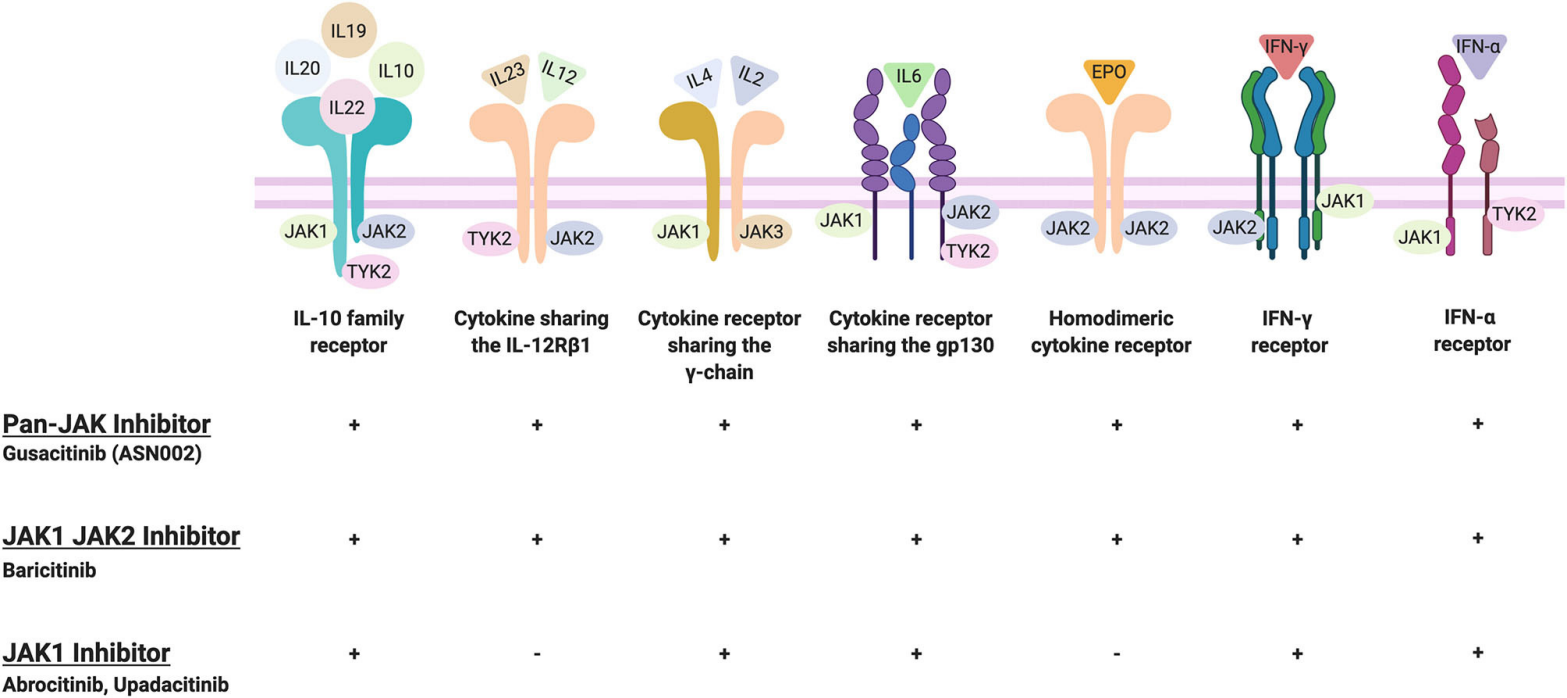
JAKi selectivity toward cytokine receptors. Created and retrieved from https://app.Biorender.com/biorender-templates.

Abrocitinib, baricitinib, and upadacitinib were the most extensively studied JAKi for the treatment of moderate-to-severe AD to date with Phase III data available. While abrocitinib and upadactinib are oral selective JAK1 inhibitors, baricitinib is an oral selective JAK1 and JAK2 inhibitor. This selectivity enables JAKi to demonstrate specificity and different capacities to block cytokine receptor signaling (Figure 3). Given that baricitinib is a JAK1/2 inhibitor, it has the capacity to inhibit the signaling of multiple cytokine receptors including the IL-10 family receptor, cytokine sharing IL-12Rß1, IFN-γ receptor, homodimeric cytokine receptor, among others. By contrast, JAK1 inhibitors can inhibit most cytokine receptors inhibited by JAK1/2 inhibitors except for the cytokine sharing IL-12Rß1 and the homodimeric cytokine receptor (Figure 3). Among the other small molecules reviewed, gusacitinib is a dual JAK/SYK inhibitor.

**Figure 4:**
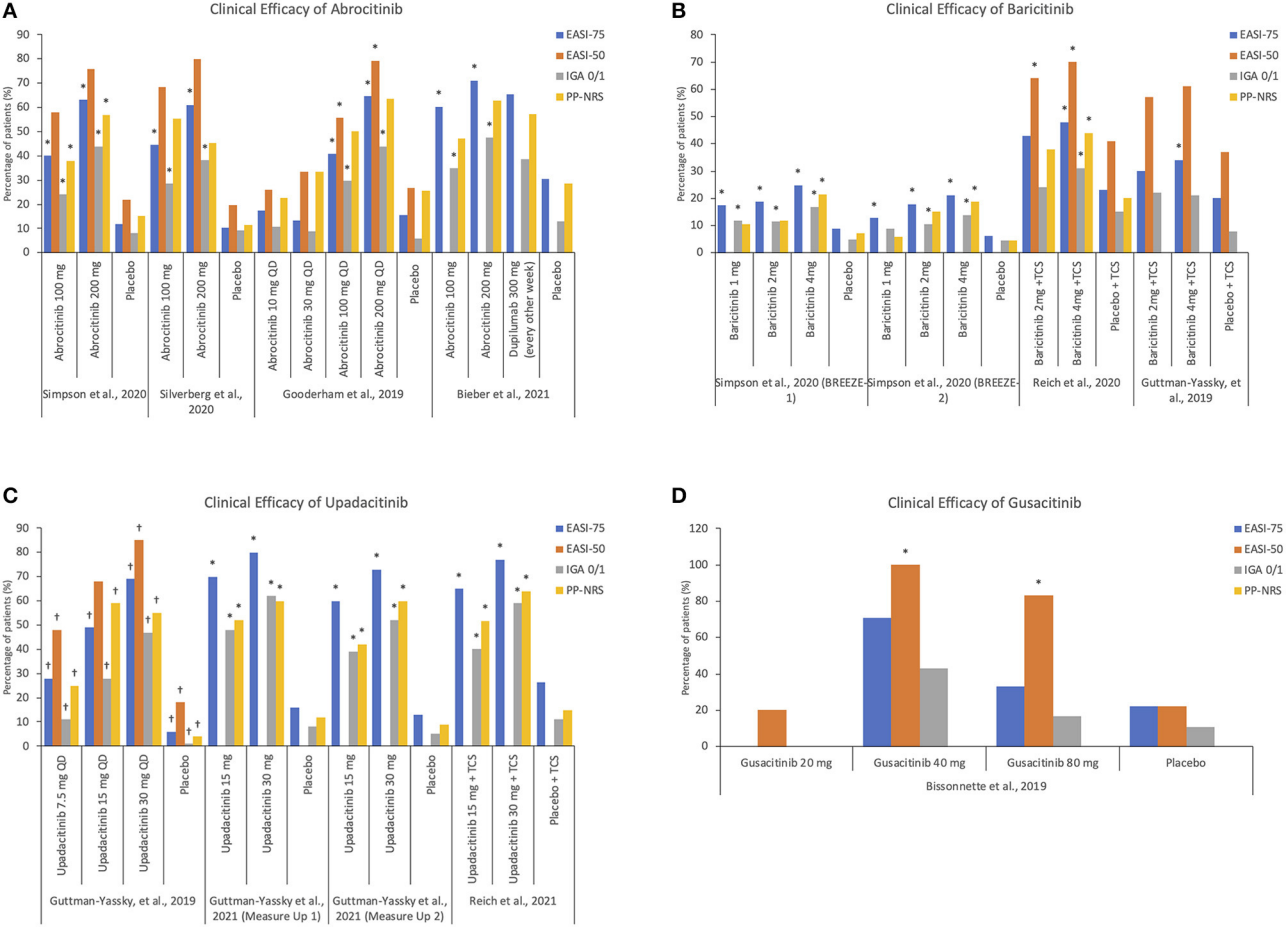
Summary of JAKi clinical efficacy by multiple outcome measures. **(A)** Clinical efficacy of abrocitinib **(B)** Clinical efficacy of baricitinib **(C)** Clinical efficacy of upadacitinib **(D)** Clinical efficacy of gusacitinib. *Values reached statistical significance; ^†^Values were estimated based on figures presented in the publication as exact values were not available. EASI, Eczema Area and Severity Index; IGA, Investigator's Global Assessment; PP-NRS, Peak Pruritus Numerical Rating Scale.

From the studies reviewed, the clinical efficacy of treatment, as defined by achieving a 4-point reduction in PP-NRS score, was achieved with 100 and 200 mg of abrocitinib, 15 and 30 mg of upadacitinib, and 4 mg of baricitinib as early as week 1 (abrocitinib and upadacitinib) and week 2 (baricitinib), respectively (33–36, 39–41). Notably, 200 mg abrocitinib dose was found to be superior to dupilimab in improving itch response at 2 weeks (39). Two mg baricitinib was also found to be effective in quickly controlling pruritus (35). This rapid efficacy is especially welcome for patients with moderate-to-severe AD, who require prompt symptom control. Similar to cyclosporine, JAKi are able to produce a fast response while demonstrating a side effect profile superior to cyclosporine. Summary of JAKi clinical efficacy by multiple outcome measures are presented in Figure 4.

The safety profile for reviewed JAKi small molecules is reassuring with most TEAE being mild and transient in nature, and amenable to symptomatic treatment. Nevertheless, it is important to note that asymptomatic increases in serum CPK levels were observed in the trials with all JAKi (34, 37, 43). These increases mirror the findings from previous studies of JAKi, including tofacitinib (47, 48), baricitinib (49), and upadacitinib (50–52), used in the treatment for a range of other inflammatory diseases. In all previous trials, CPK increases have not been associated with clinically overt myopathy or rhabdomyolysis. However, a recent case report from Australia described two patients with rheumatoid arthritis (RA), who were treated with baricitinib and developed muscle pain and joint swelling coupled to moderate CPK elevation (53). In both cases, clinical and biochemical resolution occurred rapidly after baricitinib discontinuation (53). Increases in serum lipids were also reported in response to abrocitinib and baricitinib in studies included in this review (34, 36). These findings are congruent with previous reports, particularly in response to tofacitinib (54) and baricitinib (55). Yet, while both compounds increase both LDL and HDL cholesterols, they do not appear to alter the LDL:HDL ratio (54, 56). Thus, further evaluation of cardiovascular event rates during long-term treatment is warranted to elucidate the clinical implications of these findings. Overall, it appears that the increases of CPK and lipid levels likely represent class effects, although minor differences deserving special attention in future trials might exists between JAKi compounds.

Two studies within our review reported cases of thromboembolic TEAEs after treatment with 200 mg abrocitinib or 4 mg baricitinib. In both cases, the patients had pre-existing risk factors, and one case also had a history of immobilization. Thromboembolism was first identified as a potential clinically important TEAE of JAKi during the baricitinib approval process for RA (57). In 2017, the European Medicines Agency moved to include DVT and PE as possible side effects of baricitinib, cautioning against its use in patients with risk factors for DVT or PE (58). In 2019, the Food and Drug Administration (FDA) issued a safety warning regarding the preliminary results of safety trial of tofacitinib in the treatment of RA. In patients with RA and at least one cardiovascular risk factor (CVRF), the 10 mg dose of tofacitinib given twice daily was associated with higher rates of thrombosis and all cause-mortality compared to 5 mg given twice daily or TNF-α inhibitors (59). In the absence of a mechanistic explanation for the observed increase in thromboembolic risk in patients with pre-existing CVRFs, regulatory health bodies have moved toward broadening the black box warning to include other JAKi, such as upadacitinib (which is currently approved for RA). The warning is also expected to be added to all future JAKi entering the market, including abrocitinib and gusacitinib. While there is sufficient evidence to conclude that JAKi increase the risk of thromboembolic events, it has been challenging to quantify the magnitude of this association. In one large systematic review and meta-analysis, Oliviera et al. examined the safety of JAKi in patients with inflammatory bowel disease or other immune-mediated diseases (60). Risk of DVT was assessed in 17 studies, including a total of 24,128 patients exposed to a JAKi. The overall incidence rate of VTEs was 0.31 per 100 patient-years, but the results differed between compounds. In healthy individuals, the frequency of thromboembolic events is cited at around 0.1 to 0.2 cases per 100 patient-years, increasing to about 0.5 per 100-patient-years in those aged ≥75. With such low-level frequency rates in the general population, only very large field studies could offer enough evidence for robust conclusions as to the strength of this association. Until then, JAKi should be used judiciously in patients with pre-existing cardiovascular comorbidities.

While no clinical trials within our review reported definitive treatment-related malignancies in JAKi treatment groups, a squamous cell carcinoma and keratoacanthoma were diagnosed in two patients receiving abrocitinib and updacitinib, respectively (39, 41). Nevertheless, we were unable to accurately evaluate the risk of malignancy given that these clinical trials were limited in duration. Previous studies have suggested a link between the use of tofacitinib for RA and the development of malignancies. Of 5,677 adult patients who participated in phase II, phase III and long-term extension studies of tofacitinib, 107 patients were found to develop malignancies [excluding non-melanoma skin cancer (NMSC), also known as keratinocyte carcinomas] (61, 62). The most common was lung cancer (*n* = 24), followed by breast cancer (*n* = 19), lymphomas (*n* = 10), and gastric cancer (*n* = 6). The overall incidence rate (IR) for all malignancies (excluding NMSC) in patients with RA treated with tofacitinib was 0.85 (95% CI, 0.70–1.02). Nevertheless, the incidences of all malignancies (excluding NMSC) were similar in the tofacitinib users compared with the general population (Standardized IR, 1.17; 95% CI, 0.96–1.41) (62). Consistent with this, a subsequent study following patients for 9.5 years of tofacitinib treatment documented no increased risk of malignancy in comparison to the reference population (63). Another study investigating the long-term safety of baricitinib in RA determined that incidences of malignancy (excluding NMSCs) were 0.8 (95% CI 0.4–1.5) per 100 patient-years for 2 mg baricitinib and 1.0 (95% CI 0.5–1.7) per 100 patient-years for 4 mg baricitinib, although there was no significant difference in the incidence of malignancy compared to the placebo group (64). Further long-term studies are required to appropriately determine the risk of developing malignancies when JAKi are used in AD patients. Occurrence of transient neutropenia and acne are additional important side effects to consider in the treatment with JAKi.

In conclusion, given its rapid symptom control combined with the reassuring safety profile, the use of abrocitinib, baricitinib, and upadacitinib can be considered as an important reliable systemic treatment option for adult patients with moderate-to-severe AD who are unresponsive to topical/skin-directed therapies. For abrocitinib, baricitinib, and upadacinitib, close observation of TEAEs is required as well as serial complete blood cell count with differential for neutrophil and platelet monitoring, CPK levels and lipid assessment. Hence, while needle-phobic patients may prefer an oral pill option, regular blood tests will be needed to monitor therapy. Additionally, as a clinical measure of drug efficacy, it has been hypothesized that acne as a TEAE may be due to the pharmacological effect of JAK inhibition.

Although gusacitinib has also demonstrated promising clinical outcomes in the treatment of moderate-to-severe AD, future large-scale phase III trials are required prior to considering their integration in current treatment guidelines. To the best of our knowledge, active clinical trials involving JAKi include phase III trials investigating the use of abrocitinib, baricitinib, and upadacitinib in pediatric populations. Additionally, a phase II clinical trial investigating the safety and efficacy of Jaktinib, a JAK1/2/3 inhibitor, in the treatment of moderate-to-severe AD is currently underway (ClinicalTrials.gov Identifier: NCT04612699).

JAKi represent a new therapeutic class to optimize AD treatment. As more clinical studies confirming the safety and efficacy of JAKi emerge, clinician education regarding this novel treatment will be important.

## Data Availability Statement

The original contributions presented in the study are included in the article/Supplementary Material, further inquiries can be directed to the corresponding author/s.

## Author Contributions

ML, MB-R, and FG assessed study eligibility. ML and MB-R conducted data extraction and prepared figures. ML, MB-R, FG, MB, LF, MG, LG, SH, HH, IL, PL, DM, MW, JY, CL, and IVL reviewed included studies and the extracted data and wrote the paper. CL and IVL supervised the project. All authors contributed to the article and approved the submitted version.

## Conflict of Interest

MB an advisory board member and speaker for AbbVie, Amgen, Leo Pharma, and Janssen; a speaker for Astellas and Merck; and an investigator for AbbVie, Astellas, Amgen, Leo Pharma, Novartis, Janssen, Sun Pharma, Lilly, Pfizer, and Celgene; LF an investigator for Pfizer, Amgen, Leo, and received honoraria as speaker and participant of advisory boards from the same companies. She is also a speaker for Pierre Fabre and Galderma; MG has been an investigator, speaker or advisory board member for Abbvie, Akros, Amgen, Arcutis, Boehringer Ingelheim, BMS, Celgene, Dermira, Dermavant, Galderma, GSK, Eli Lilly, Incyte, Janssen, Kyowa Kirin, Leo Pharma, Medimmune, Merck, Novartis, Pfizer, Regeneron, Sanofi Genzyme, Sun Pharmaceuticals, UCB, Valeant/Bausch; LG has been a consultant, advisory board member, speaker and clinical trials principal investigator for AbbVie, Eli Lilly, LaRoche Posay, Leo Pharma, Pfizer and Roche, and a consultant, advisory board member and speaker for Bausch Health, Johnson & Johnson, and Sanofi Aventis. SH has been a consultant and/or advisor and/or investigator for or received honoraria from Abbvie, Akros, Altius Healthcare, Amgen, Aralez, Arcutis, Bausch Health, Biopharma, BMS, Boehringer-Ingelheim, Celgene, Coherus, Concert Pharma, Cutanea, Dermira, Galderma, Glenmark, Incyte, Janssen, Leo, Lilly, Novartis, Pedia-Pharm, Pfizer, Regeneron, Sandoz, Sanofi, Sun Pharma, UCB; HH has been a consultant and/or advisor and/or investigator for or received honoraria from Abbvie, Amgen, Arcutis, Bausch Health, Boerhinger Ingelheim, Bristol Meyers Squibb, Celgene, Dermavant, Dermira, DS Biopharma, Eli Lilly, Galderma, GlaxoSmithKline, Incyte, Janssen, Leo Pharma, Medimmune, Merck, Novartis, Pfizer, Regeneron, Sanofi, Sun Pharma, and UCB; PL has been a consultant and/or advisor and/or investigator for or received honoraria from Abbvie, Amgen, Aralez, Bausch Health, Boehringer-Ingelheim, Celgene, Dermira, Galderma, Janssen, Leo, Lilly, Novartis, Pfizer, Regeneron, Sanofi, Sun Pharma; DM has been a consultant and/or investigator and/or speaker for Abbvie, Celgene, Janssen, Leo Pharma, Lilly, Novartis, Pfizer, Regeneron, Sanofi, UCB; MW reports having received honoraria for ad board participation from Novartis, Sun Pharma and Pfizer; JY has been a speaker, consultant, and investigator for AbbVie, Allergan, Amgen, Arcutis, Astellas, Bausch Health, Boehringer Ingelheim, Bristol Meyers Squibb, Celgene, Centocor, Coherus, Dermavant, Dermira, Forward, Galderma, GlaxoSmithKline, Incyte, Janssen, Kyowa, Leo Pharma, Lilly, Medimmune, Merck, Novartis, Pfizer, Regeneron, Roche, Sandoz, Sanofi Genzyme, Sun Pharma, Takeda, UCB, and Xenon. CL was a consultant, speaker, and advisory board member for Amgen, Pfizer, AbbVie, Janssen, Novartis, and Celgene, and was an investigator for Amgen, Pfizer, AbbVie, Janssen, Lilly, Novartis, and Celgene. Dr Poulin was a speaker and advisory board member for AbbVie, Amgen, and Janssen, and was an investigator for AbbVie, Amgen, Celgene, Centocor, Lilly, Galderma, Incyte, Sun Pharma, Janssen, Leo Pharma, Merck, Novartis, Pfizer, and Roche; IVL received grant funding from Novartis, Merck, AbbVie, and Bristol Myers Squibb and honoraria from Janssen, Bausch, Galderma, Novartis, Pfizer, Sun Pharmaceuticals, Johnson & Johnson, and Actelion. The remaining authors declare that the research was conducted in the absence of any commercial or financial relationships that could be construed as a potential conflict of interest.

## Publisher's Note

All claims expressed in this article are solely those of the authors and do not necessarily represent those of their affiliated organizations, or those of the publisher, the editors and the reviewers. Any product that may be evaluated in this article, or claim that may be made by its manufacturer, is not guaranteed or endorsed by the publisher.
