# Supplementary material for: Systematic Review on the Efficacy and Safety of Oral Janus Kinase Inhibitors for the Treatment of Atopic Dermatitis
Source: Front Med (Lausanne). 2021 Sep 1;8:682547. doi: 10.3389/fmed.2021.682547 (PMC8440866; doi:10.3389/fmed.2021.682547)
Supplement: Supplementary file 1 [file Data_Sheet_1.docx]

**Supplementary Material**

**Supplementary Text 1: Search strategies by study database**

**EMBASE**

#1 ("atopic dermatitits" or "atopic eczema" or "dermatitis, atopic " or "dermatitis, atopic" or "eczema atopica" or "eczema endogenous" or "eczema infantum" or "eczema, infantile" or "endogenous eczema" or "infantile eczema" or "neurodermatitis constitutionalis" or "neurodermatitis disseminata" or "neurodermatitis, atopic constitutional").mp. or "atopic dermatitis"/ use oemezd

#2 ("JAK inhibitor" or "jak inhibitor" or "janus kinase inhibitors " or "janus tyrosine kinase inhibitor").mp. or exp "Janus kinase inhibitor"/ use oemezd

#3 1 AND 2

#4 Limit 3 to humans only

**MEDLINE**

#1: ("atopic dermatitits" or "atopic eczema" or "dermatitis, atopic " or "dermatitis,atopic" or "eczema atopica" or "eczema endogenous" or "eczema infantum" or "eczema, infantile" or "endogenous eczema" or "infantile eczema" or "neurodermatitis constitutionalis" or "neurodermatitis disseminata" or "neurodermatitis,atopic constitutional").mp. or exp "Dermatitis, Atopic"/ use medal

#2: "JAK inhibitor" or "inhibitors, jak" or "inhibitors, janus kinase" or "jak inhibitors" or "janus kinase inhibitors" or "kinase inhibitors, janus").mp. or exp "Janus Kinase Inhibitors"/ use medall

3) 1 AND 2

4) Limit 3 to humans only

**Pubmed**

#1: "JAK inhibitor"[All Fields] OR "JAK inhibitor"[All Fields] OR "janus kinase inhibitors"[All Fields] OR "janus tyrosine kinase inhibitor"[All Fields]

#2: (("atopic"[All Fields] OR "atopical"[All Fields] OR "atopics"[All Fields]) AND "dermatitits"[All Fields]) OR "atopic eczema"[All Fields] OR "dermatitis,atopic"[All Fields] OR "dermatitis,atopic"[All Fields] OR (("eczema"[MeSH Terms] OR "eczema"[All Fields] OR "eczemas"[All Fields]) AND ("atopica"[All Fields] OR "cyclosporine"[MeSH Terms] OR "cyclosporine"[All Fields] OR "ciclosporin"[All Fields] OR "ciclosporine"[All Fields] OR "cyclosporin"[All Fields] OR "cyclosporine s"[All Fields] OR "cyclosporine"[MeSH Terms] OR "cyclosporins"[All Fields] OR "cyclosporines"[All Fields])) OR (("eczema"[MeSH Terms] OR "eczema"[All Fields] OR "eczemas"[All Fields]) AND ("endogen"[All Fields] OR "endogene"[All Fields] OR "endogeneous"[All Fields] OR "endogeneously"[All Fields] OR "endogenes"[All Fields] OR "endogenic"[All Fields] OR "endogenous"[All Fields] OR "endogenously"[All Fields])) OR "eczema infantum"[All Fields] OR "eczema infantile"[All Fields] OR "endogenous eczema"[All Fields] OR "infantile eczema"[All Fields] OR (("neurodermatitis"[MeSH Terms] OR "neurodermatitis"[All Fields]) AND "constitutionalis"[All Fields]) OR "neurodermatitis disseminata"[All Fields] OR (("dermatitis, atopic"[MeSH Terms] OR ("dermatitis"[All Fields] AND "atopic"[All Fields]) OR "atopic dermatitis"[All Fields] OR ("neurodermatitis"[All Fields] AND "atopic"[All Fields]) OR "neurodermatitis atopic"[All Fields]) AND ("constitution and bylaws"[MeSH Terms] OR ("constitution"[All Fields] AND "bylaws"[All Fields]) OR "constitution and bylaws"[All Fields] OR "constitution"[All Fields] OR "constitutions"[All Fields] OR "constitutional"[All Fields]))

#3: ("JAK inhibitor"[All Fields] OR "JAK inhibitor"[All Fields] OR "janus kinase inhibitors"[All Fields] OR "janus tyrosine kinase inhibitor"[All Fields]) AND ((("atopic"[All Fields] OR "atopical"[All Fields] OR "atopics"[All Fields]) AND "dermatitits"[All Fields]) OR "atopic eczema"[All Fields] OR "dermatitis,atopic"[All Fields] OR "dermatitis,atopic"[All Fields] OR (("eczema"[MeSH Terms] OR "eczema"[All Fields] OR "eczemas"[All Fields]) AND ("atopica"[All Fields] OR "cyclosporine"[MeSH Terms] OR "cyclosporine"[All Fields] OR "ciclosporin"[All Fields] OR "ciclosporine"[All Fields] OR "cyclosporin"[All Fields] OR "cyclosporine s"[All Fields] OR "cyclosporins"[MeSH Terms] OR "cyclosporins"[All Fields] OR "cyclosporines"[All Fields])) OR (("eczema"[MeSH Terms] OR "eczema"[All Fields] OR "eczemas"[All Fields]) AND ("endogen"[All Fields] OR "endogene"[All Fields] OR "endogeneous"[All Fields] OR "endogeneously"[All Fields] OR "endogenes"[All Fields] OR "endogenic"[All Fields] OR "endogenous"[All Fields] OR "endogenously"[All Fields])) OR "eczema infantum"[All Fields] OR "eczema infantile"[All Fields] OR "endogenous eczema"[All Fields] OR "infantile eczema"[All Fields] OR (("neurodermatitis"[MeSH Terms] OR "neurodermatitis"[All Fields]) AND "constitutionalis"[All Fields]) OR "neurodermatitis disseminata"[All Fields] OR (("dermatitis, atopic"[MeSH Terms] OR ("dermatitis"[All Fields] AND "atopic"[All Fields]) OR "atopic dermatitis"[All Fields] OR ("neurodermatitis"[All Fields] AND "atopic"[All Fields]) OR "neurodermatitis atopic"[All Fields]) AND ("constitution and bylaws"[MeSH Terms] OR ("constitution"[All Fields] AND "bylaws"[All Fields]) OR "constitution and bylaws"[All Fields] OR "constitution"[All Fields] OR "constitutions"[All Fields] OR "constitutional"[All Fields])))

**Supplementary Table 1.** Quality assessment table

| Trial ID | Bias from randomization process | Bias due to deviations from intended intervention | Bias due to missing outcome data | Bias in the measurement of the outcome | Bias in the selection of reported results | **Overall bias.** | Comments |
| --- | --- | --- | --- | --- | --- | --- | --- |
| Simpson ^a, b^ et al., 2020 | Low | Low | Low | Low | Low | Low | High attrition rates within groups. However, reasons for missing outcome data do not differ substantially between intervention groups, thus we deemed risk of bias as low. |
| Silverberg ^a, b^ et al., 2020 | Low | Low | High | Low | Low | High | Attrition rates and reasons for missing outcome data differ between groups by factors that are directly related to primary outcome: in placebo group, patients were more likely than tx groups to discontinue due to AEs, being lost to f/u or no-longer willing to participate, insufficient clinical response and for protocol violation. |
| Bieber^a^ et al., 2021 | Low | Low | Low | Low | Low | Low | Approximately 10% attrition rate for each group, with similar reasons for discontinuation reported across groups. |
| Gooderham ^a, b, c^ et al., 2019 | Low | Low | High | Low | Low | High | Significant differential attrition rates between study groups (50% in placebo, Abrocitinib: 45% in 10 mg, 46% in 30 mg, 33% in 100 mg and 30% in 200 mg groups). Reasons provided differ between groups. Since mITT analysis, bias likely towards the null. |
| Simpson ^d^ et al., 2020 | Low | Low | Low | Low | Low | Low | Approximately 10% attrition rate for each group, with similar reasons for discontinuation reported across groups. |
| Reich ^d^ et al., 2020^36^ | Low | Low | Low | Low | Low | Low | All patients received moderate- and/or low potency TCSs (such as 0.1% triamcinolone cream and 2.5% hydrocortisone ointment, respectively) for active lesions ^d^. |
| Guttman-Yassky ^e^ et al., 2019 | Low | Low | High | Low | Low | High | Significant differential attrition rates between study groups: 41% placebo, 32% in 2 mg Baricitinib, and 32% in the 4 mg Baricitinib group did not complete study. Since ITT analysis, bias likely towards the null. |
| Guttman-Yassky et al., 2021 | Low | Low | Low | Low | Low | Low | Approximately 10% attrition rate for each group, with similar reasons for discontinuation reported in intervention groups. Placebo had more cases due to lack of efficacy |
| Reich et al., 2021 | Low | Low | Low | Low | Low | Low | Approximately 5% attrition rate for each group, with similar reasons for discontinuation reported in intervention groups. |
| Guttman-Yassky et al., 2020 | Some concerns | Low | High | Low | Low | High | Differences between groups in baseline severity of AD as measured by IGA. Significant differential attrition rates between study groups in reason for discontinuing study (placebo more cases due to lack of efficacy). |
| Bissonnette et al., 2019 | High | High | High | Low | Low | High | Imbalances between tx groups baseline characteristics in severity of disease based on IGA score. Per-protocol-analysis. Attrition differs between tx groups, with greater number of participants in placebo discontinuing due to protocol non-compliance and more in tx groups related to pre-determined stopping-rules^f^. |

*Note.* mITT- modified intervention-to-treat analyses; TCS- topical corticosteroids; AD- Atopic Dermatitis; Tx -treatment; f/u - follow-up; IGA- Investigator’s Global Assessment.

^a^ Efficacy assessed via a modified ITT that includes patients as randomized who had received ≥ 1 dose of experimental drug.

^b^ Patients able to use antihistamines and non-medicated emollients throughout study.

^c^ Efficacy assessed via a modified ITT that includes patients as randomized who had received ≥ 1 dose of experimental drug except 4 patients excluded for major protocol deviations secondary to lack of principal investigator oversight.

^d^ Although systemic and topical treatments were allowed as rescue tx if the patients experienced worsening or unacceptable AD symptoms, efficacy analyses were censored at point of drug discontinuation or at point when rescue tx was introduced.

^e^ Triamcinolone (0.1%) was used throughout the study according to labelling or as recommended by investigator.

^f^ Events meeting stopping rules included mild HTN and low lymphocyte levels.
